# Maintenance of Deep Lung Architecture and Automated Airway Segmentation for 3D Mass Spectrometry Imaging

**DOI:** 10.1038/s41598-019-56364-4

**Published:** 2019-12-27

**Authors:** Alison J. Scott, Courtney E. Chandler, Shane R. Ellis, Ron M. A. Heeren, Robert K. Ernst

**Affiliations:** 1 Department of Microbial Pathogenesis, University of Maryland School of Dentistry, Baltimore, 21201 USA; 2 Maastricht Multimodal Molecular Imaging (M4I) Institute, Maastricht University, Maastricht, 6229ER Netherlands

**Keywords:** Lipidomics, Molecular medicine

## Abstract

Mass spectrometry imaging (MSI) is a technique for mapping the spatial distributions of molecules in sectioned tissue. Histology-preserving tissue preparation methods are central to successful MSI studies. Common fixation methods, used to preserve tissue morphology, can result in artifacts in the resulting MSI experiment including delocalization of analytes, altered adduct profiles, and loss of key analytes due to irreversible cross-linking and diffusion. This is especially troublesome in lung and airway samples, in which histology and morphology is best interpreted from 3D reconstruction, requiring the large and small airways to remain inflated during analysis. Here, we developed an MSI-compatible inflation containing as few exogenous components as possible, forgoing perfusion, fixation, and addition of salt solutions upon inflation that resulted in an ungapped 3D molecular reconstruction through more than 300 microns. We characterized a series of polyunsaturated phospholipids (PUFA-PLs), specifically phosphatidylinositol (-PI) lipids linked to lethal inflammation in bacterial infection and mapped them in serial sections of inflated mouse lung. PUFA-PIs were identified using spatial lipidomics and determined to be determinant markers of major airway features using unsupervised hierarchical clustering. Deep lung architecture was preserved using this inflation approach and the resulting sections are compatible with multiple MSI modalities, automated interpretation software, and serial 3D reconstruction.

## Introduction

Mass spectrometry imaging (MSI) is a molecular histology technique currently used for biomarker discovery, drug distribution, and disease characterization^1–3^. In brief, an MSI experiment consists of rastering a desorption/ionization probe across a tissue section and detecting the generated ions from each location using mass spectrometry^4,5^. MSI does not rely on tagged or labeled reagents (antibodies or probes) and does not depend on any *a priori* species-specific knowledge making MSI a flexible tool for discovery^6–9^. Ionization methods for MSI have been well reviewed, including the most common method matrix-assisted laser desorption/ionization (MALDI)^10–12^. Tissue preparation for MALDI-MSI begins with depositing a matrix substance evenly across the tissue using sublimation or a solvent spray^13,14^. In its various embodiments, the spatial resolution of a MALDI-MSI experiment can vary between 1 and 100 microns^15,16^, though larger tissues and higher-throughput experiments are commonly performed at lower resolutions, between 50 and 100 microns. Several molecular classes are routinely detected by MALDI-MSI including small intact proteins^2^, peptides (after on-tissue digestion)^17^, metabolites^18^, glycans^19^, and lipids^12,20,21^.

Since the underlying sample in an MSI study is a thin tissue section (typically 10–15 µm thickness), preservation of histological structures during sample preparation is paramount to accurate data interpretation. For most solid or semi-solid tissues, maintaining spatial organization during cryosectioning and slide mounting is straightforward, unlike tissues with large luminal areas, such as the lung and gastrointestinal tract. Lung tissue needs to be well-fixed or have the airspaces filled with material prior to dissection to maintain the tissue architecture and functional space that normally exists as a result of the negative pressure applied by the diaphragm. Further considerations are necessary in preparation for MSI experiments involving lung tissue. To achieve high quality histology from rodent airways, the tissue must be inflated from the trachea to maintain open bronchoalveolar architecture. For samples intended for a range of readouts from basic histological stains to advanced micro-X-ray CT analysis, lung tissue can be first preserved by formalin fixation followed by paraffin-embedding (FFPE) and sectioning before analysis^22^. Alternatively, the lung can be filled with a solution of fixative with matrix solution, such as agarose before sectioning and analysis^23^. In contrast, rodent lungs intended for cryosectioning in preparation for immunostains and probe readouts are commonly prepared by inflation via the trachea using a solution of cryoembedding media mixed with neutral buffer to decrease the viscosity of the solution. Cryoembedding media (typically Optimal Cutting Temperature compound, OCT) consists of mixed polymers and preservatives that are usually avoided for downstream molecular applications such as an MSI experiment^24^. While FFPE prepared tissues are compatible with some MSI readouts, it is not compatible with lipid profiling due to the paraffin removal steps and the potential loss or modification of key lipid classes upon fixaton^5^. Using modified OCT (mOCT) compound, Zemiki-Berry, *et al*. reported a MSI-compatible rodent lung inflation method that identified major classes of lipids in both the positive and negative ion modes associated with histological features (large airway, vasculature, etc.) in single tissue sections^25^. A key finding of this study was the association of phospholipids containing polyunsaturated fatty acids (PUFAs) at the lining of airway structures^25^. Using MALDI-FTICR, Jones, *et al*. inflated mouse lungs using a solution of carboxymethylcellulose (CMC) resulting in the creation of a high mass resolution, tissue-spanning 3D reconstruction of a lung with voxel dimensions of 120 × 120 × 120 μm, demonstrating that 3D reconstruction of gapped serial sections is possible in this delicate tissue^26^.

In this work, we developed and demonstrated an alternative inflation medium (porcine gelatin) that preserves lung ultrastructure and is compatible with advanced MSI techniques including three-dimensional reconstruction of fine-scale consecutive serial sections, spatial segmentation analysis, and lipid profiling using parallelized lipidomic imaging. Preservation of histology in lung tissues for MSI experimentation is a priority for distribution studies of inhaled drug products, lung infections, chronic lung dysfunctions, and cancers. These advances in preparation techniques, as well as in histological interpretation using unique lipid signatures, contribute to the ongoing development of a spatially-resolved mouse lung lipidome.

## Materials and Methods

### Mice

Adult, female C57BL/6J mice (Jackson Laboratories, Bar Harbor, ME) were housed in a specific pathogen free, particle-filtered negative pressure airflow caging system. Food and water were provided *ad libitum*. Mice were euthanized by carbon dioxide narcosis followed by thoracotomy during the inflation procedure. All animal procedures were performed under an approved protocol administered by the Institutional Animal Care and Use Committee at the University of Maryland - Baltimore.

### Lung inflation

Post-mortem, mice were pinned to a dissection board, the fur wetted with 70% ethanol, and the skin was opened along the midline from the mid-abdomen to the jawline to cleanly expose the thorax. Next, the muscle layer was opened exposing the liver and diaphragm, the liver was pulled toward the stomach and the diaphragm was cut, deflating the lungs. The thoracic cavity was prepared by removing the entire ventral rib cage, cutting near the line formed by the intersection of the ventral/dorsal rib segments, taking care not to disturb the heart, lungs, or trachea. The salivary glands were removed, fully exposing the trachea. For these studies, the thymus was removed prior to lung inflation, although optional. A sterile, 5 mL Leur-lock syringe was filled with 2 mL of a solution of 2% porcine gelatin in molecular-grade water cooled before use. Two lengths of suture (4–0 Vicryl Braided, Ethicon, Sommerville, NJ) were threaded under the trachea approximately 2–3 mm apart and approximately 2 mm from the larynx. Both sutures were loosely tied with a double-twisted single knot. A catheter needle (Jelco 20G1-1/4 IV catheter, Smiths Medical, Minneapolis, MN) with a Leur-lock fitting was inserted into the trachea between the larynx and the first suture. When the catheter tip reached the tracheal interior, the needle was retracted to prevent perforation and the catheter was gently guided into the trachea below the second suture. The suture knots were subsequently tightened to secure the catheter. The syringe containing the gelatin solution was affixed to the Leur end of the catheter and the lungs inflated with ~1–1.5 mL of gelatin solution. The inflation was complete when the distal fringes of the lungs inflate, especially the accessory lobe. Subsequently, the trachea and catheter tube was secured, the catheter tube was pinched by twisting to prevent backflow, and the trachea cut using forceps. Any remaining connections to the spine were gently cut away and the inflated lungs and heart were lifted out of the pleural cavity. The descending aorta and inferior vena cava were cut and the lungs (dorsal side down) were placed onto a small aluminum foil square (approximately 2.5 cm × 2.5 cm), floated on a pool of liquid nitrogen and allowed to freeze for at least 2 minutes. Frozen, inflated lungs were stored in a foil envelope inside sealed plastic storage bags at −80 °C until analysis. All reagents were sourced from Sigma-Aldrich (St. Louis, MO) unless otherwise noted.

### Sectioning gelatin inflated lungs

A Leica CM1950 cryomicrotome (Leica, Buffalo Grove, IL) was pre-equilibrated to a chamber and blade temperature of −12 °C. Inflated lungs were mounted to the cryostage using cooled, molecular biology grade water, dorsal side down. The mounted lungs equilibrated to cabinet temperature for at least 20 minutes. Trim sections of 40–50 μm were discarded until reaching the depth of a major airway feature on either the left or right side. Sections were cut at this depth at 13 μm section thickness and thaw-mounted at 37 °C to a pre-cooled indium tin oxide (ITO; Delta Technologies, Loveland, CO) slide until visibly dry. Slide-mounted tissues were desiccated for 20 minutes and stored at −80 °C or used immediately for imaging experiments.

### Matrix application for lipid imaging

The dual-polarity lipid imaging matrix, norharmane (NRM)^21,27^ was prepared at 7 mg/mL in a solution of 2:1 chloroform:methanol (v:v). The NRM solution was applied using an HTX TM-sprayer (HTX Technologies, Chapel Hill, NC) using the following settings: 30 °C nozzle temperature, 10 passes, 0.120 mL/min flow rate, 1200 mm/min velocity, 3 mm track spacing, 10 psi pressure, 3 L/min gas flow, 30 s dry time between passes, using the “CC” coverage pattern. Following matrix application, slides were desiccated for at least 20 minutes prior to MSI.

### Negative ion mode lipid imaging

All images for 3D reconstruction were captured on a Bruker rapifleX MALDI-TOF/TOF (Bruker Daltonics, Bremen, DE) in negative ion mode. The instrument was calibrated using red phosphorus clusters spotted on each individual slide. Calibration was performed prior to analyzing each individual section. For each tissue, minor adjustments were made to the laser power to yield less than 100,000 arbitrary intensity of the base peak in the spectrum (on tissue) to account for slight variations in tissue height. Each tissue was analyzed with the following capture settings: *m/*z range 600–1700, 200 shots/pixel, rastering at 50 μm, in negative reflectron mode. All images were normalized to total ion current (TIC).

### 3D Reconstruction of negative mode lipid images

Lung samples were prepared as above from untreated, naïve mice. Serial sections were collected as above from 24 consecutive tissue slices at 13 μm from the center mass of the tissue. Each tissue section was imaged exactly as described in the *Lipid Imaging* section in negative ion mode using NRM matrix as above. Imaging data was collected on a Bruker rapiFlex MALDI-TOF instrument using 50 μm rastering (as above). Data collection for each tissue section was completed in approximately 25–30 minutes using a Bruker rapiFlex totaling twelve hours of active data collection time. Data from all sections were imported, normalized to TIC, and spatially-aligned using SCiLS 2016b 3D software (SCiLS Lab, Bremen, Germany)^28^. Section-to-section co-registration was performed on prominent features such as the ventricle chamber and large airway inflection points. Video renderings (as.png files) were assembled in a 360° horizontal and vertical rotation view at 10 seconds time lapse.

### Segmentation analysis of inflated lungs

MALDI-TOF imaging data from one section (#21) of the serial set was arbitrarily chosen for segmentation analysis. The negative ion mode data was loaded into SCiLS Lab version 2016b (SCiLS Lab, Bremen, Germany) and normalized to TIC. The automated SCiLS segmentation pipeline was used to determine and visualize the spatially defined spectral components of the inflated lung section. Spectral data were exported as.CSV files and imported into mMASS software^29^ for figure preparation.

### High mass resolution imaging and tandem mass spectrometry of inflated lung

For identification of the detected lipid species, a portion of an inflated lung lobe was chosen for analysis using the data dependent acquisition (DDA)-imaging method, as previously described^30^. Data was acquired using an Orbitrap Elite mass spectrometer (Thermo Fisher Scientific GmbH, Bremen, Germany) coupled to a dual MALDI/ESI interface (Spectroglyph LLC, Kennewick, CA, USA)^31^. In this approach, high-mass resolution Orbitrap scans used for MSI and accurate *m/z* determination (*m/*z range 180–2000, mass resolution = 240,000 @ *m/z* 400) and data-dependent ion trap MS/MS using collision-induced dissociation (±0.5 Da isolation window and normalized collision energy of 40) were acquired in parallel at adjacent sampling positions across the tissue surface. The stage step size 20 µm × 40 µm resulted in an MSI pixel size of 40 µm × 40 µm. The analyzed area was selected based on the presence of all of the following in one area: endothelial structures, large and small airway features, and abundant lung parenchyma surroundings. Imaging was performed in negative ion mode using NRM matrix applied as above using an ion injection time for both Orbitrap and ion-trap MS/MS scans of 250 ms with automatic gain control turned off and a laser repetition rate repetition rate of 1 kHz. Image reconstruction and visualization was performed according to previously published methods^30^. Prior to acquisition the instrument was calibrated using the standard calibration mixture with electrospray ionization.

### Staining of tissue post-MSI

Matrix-coated slides with analyzed sections were stained by hematoxylin and eosin as follows. Briefly, matrix was removed by dipping in a bath of 50% methanol. After matrix was visibly removed, the slides were dipped for 30 seconds in 70% ethanol then submerged in a Hematoxylin bath (Gill’s #2) and let to stain for 2 minutes. Hematoxylin stained slides were rinsed in a bath of running deionized water until the water rinsed clear from the slide. Bluing was done by dipping the slides into a solution of 1% ammonium hydroxide in tap water and immediately submerging in acidified eosin stain for 2 minutes. Following eosin staining slides were rinsed in successive washes of 70% ethanol until the rinsate ran clear. Slides were dehydrated by incubating for 2 minutes each in increasing percentages of ethanol (80, 90, 95, 100). Sections were cleared by dipping in xylenes or Citri-solv prior to coverslipping (Corning Glass, Corning, NY) mounting with Permount. All stains and mounting reagents were purchased from Sigma-Aldrich (St. Louis, MO) and solvents were sourced from Fisher Scientific (Thermo Fisher Scientific, Waltham, MA).

## Results

### Gelatin inflation preserves lung histology for MSI

**Figure 1:**
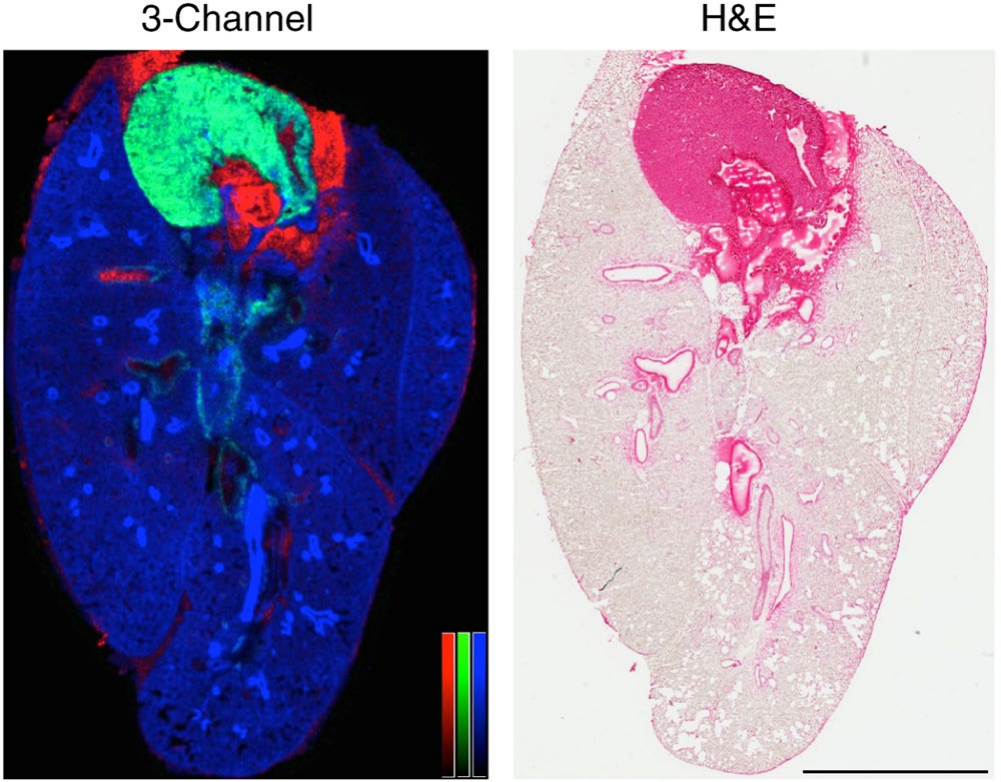
Gelatin inflation of the lung prior to MSI preserves overall lung histology. Mouse lung was inflated with gelatin and imaged by MALDI-TOF in negative ion mode (50 μm spatial resolution). Left: tricolor reconstruction of heme (red, *m/z* 615.1), cardiolipin (green, *m/z* 1447.8), phosphatidylinositol [PI(38:4)-H]^−^ (blue, *m/z* 885.5), normalized to TIC. Right: H&E staining of section, post-MSI. Color bars = 0–100% relative intensity. Scale bar = 5 mm.

The purpose of lung inflation prior to imaging is to maintain *in vivo* tissue architecture and fine structure. To evaluate the preservation of lung architecture using MSI, we prepared a section of gelatin inflated mouse lung for negative ion mode lipid imaging by high-speed MALDI-TOF. Since the inflation method is performed with the heart and lungs intact, the sections contained multiple lung lobes with large airway structures, the heart with a ventricle chamber, and blood in the vasculature structures. One of the major aims of our study was to develop a reproducible working method to create serial sections for MSI through at least 200 μm of tissue thickness for fine-scale 3D molecular reconstructions. Lower percentages of gelatin in water made the tissue sections too brittle and tissue crumbled on the cutting blade. In contrast, higher percentages of gelatin yielded samples that were too soft and the tissue was distorted by mild smearing on the cutting blade and repeated serial section could not be collected without distortion for high fidelity image alignment. In our testing, other inflation compositions (OCT mixtures and CMC mixtures) also yielded troublesome serial sectioning. The protocol of 2% gelatin inflation resulted in acceptable serial sections in every tissue tested ( > 30 independent tissue preparations). To evaluate the resulting histology in gelatin inflated lung samples, we first reconstructed the basic section architecture using characteristic markers of these three compartments and compared them to an H&E stain of the same section (post-MSI) (Fig. 1). The cardiolipin (CL) species CL(72:8) was observed as the [CL(72:8)-H]^−^ ion detected at *m/z* 1447.8. CL is a major component of cardiac tissue^32^, owing to the abundance of mitochondria in the heart (up to 40% of cellular volume^33^). Heme was detected as a negative ion at *m/z* 615.1 and was used to map blood in the heart chambers, lung vasculature, and localized bleeding from the excision procedure. Finally, a PUFA-PI, [PI(18:0_20:4)-H]^−^ ion at *m/z* 885.5, predominantly composed of stearoyl, arachidonyl fatty acyl chains, i.e - SAPI), was used to demarcate the airway linings (in areas of high ion intensity) and fill in the lung parenchyma (at areas of low-to-moderate ion intensity). SAPI was previously implicated in the lethal eicosanoid storm in mice infected with the bacterial species *Francisella novicida*, owing to the accumulation of lipid precursors upstream of prostaglandin E2 (PGE_2_) synthesis^27^. The overall tissue architecture was accurately reconstructed at 50 μm spatial resolution using these three ions (*m/z* 1447.8 - green, 615.1 - red, and 885.5 - blue), representing the heart tissue, blood, and airway structures, respectively (Fig. 1).

**Figure 2:**
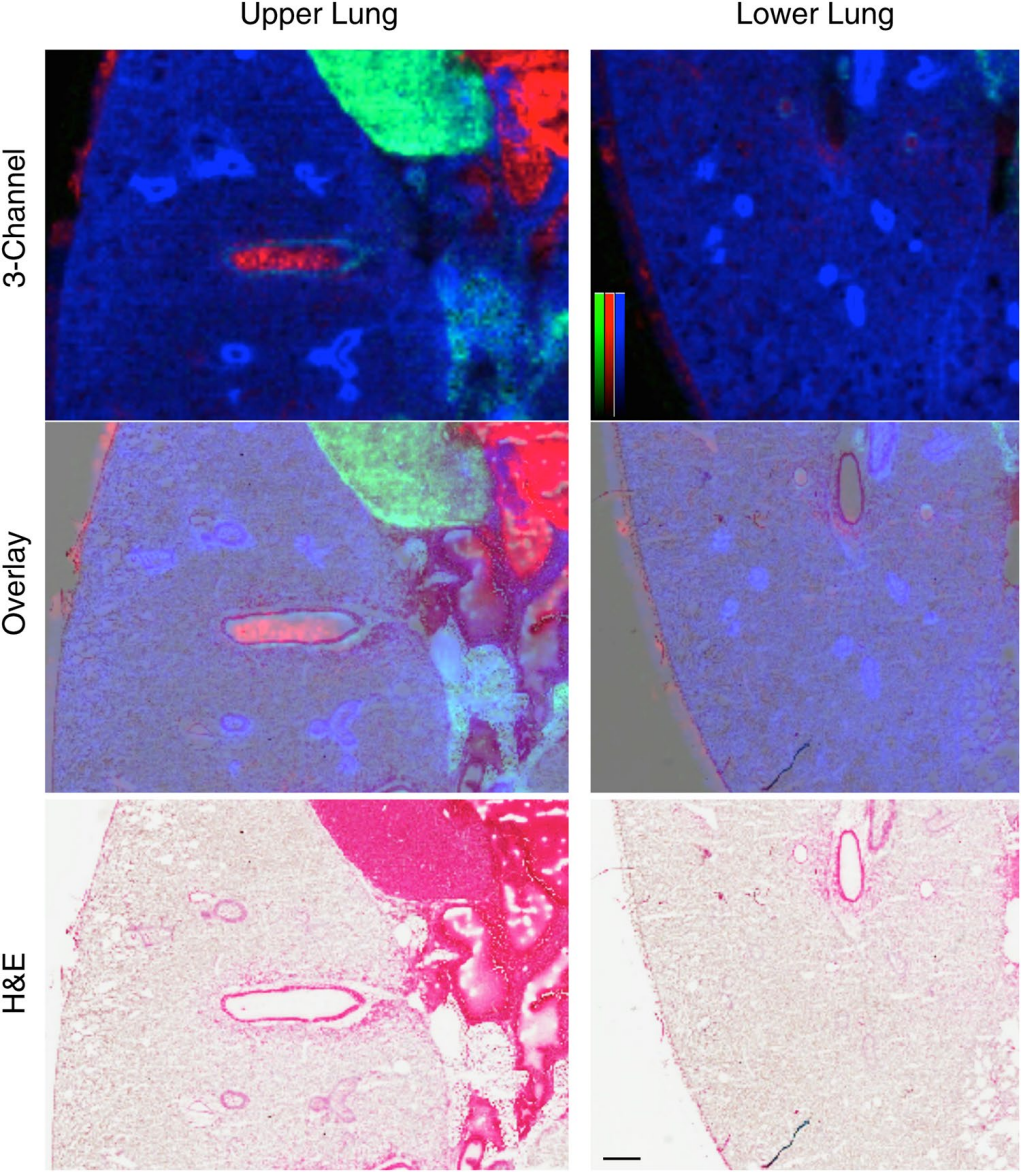
Deep lung fine structures are preserved edge-to-edge in gelatin inflated lungs. Mouse lung, gelatin inflated, MALDI-TOF in negative ion mode (50 μm spatial resolution).Left panel: upper lung, with heart in view; Right panel: lower lung with intact, continuous serosa edge. Top panel: 3-channel (tricolor) reconstruction of heme (red, *m/z* 615.1), cardiolipin (green, *m/z* 1447.8), phosphatidylinositol [PI(38:4)-H]^−^ (blue, *m/z* 885.5), normalized to TIC. Bottom: H&E staining of section, post-MSI. Middle: overlay of tricolor image and H&E. Color bars = 0–100% relative intensity. Scale bar = 500 μm.

Compared to the underlying H&E stain, the MSI characteristics defined by these three ions accurately match the tissue components on a gross examination level. In addition, the MSI image also accurately represents the airway and endothelial structures, as well as the surrounding tissue. At 50 μm spatial resolution, the airway and vascular linings are well resolved, however the MSI image is not fine enough to resolve individual alveolar structure (mean airspace chord length of an adult C57BL/6 mouse alveolus is approximately 40–50 μm^34^). In a magnified view shown in Fig. 2, the MSI image is still representative of histological structures using the same ions identified in Fig. 1. Notably, the distal lung shows open alveolar spaces by H&E, both the open airways of the upper and lower lungs are preserved, and the visceral pleura is intact. The tissue architecture in gelatin inflated lung sections with MSI and without MSI is both similar to traditionally prepared FFPE prepared and H&E stained sections (Fig. S1 and **Supplemental Information**).

The baseline spectrum resulting from 2% gelatin (prepared for imaging with NRM matrix) was evaluated and compared to other reported lung inflation mixtures (**Supplemental Information**). In positive mode, no interfering ions were detected in the phospholipid range in any of the tested conditions (50% OCT in water, 50% OCT in PBS, 1% CMC in water, 2% gelatin in water) (Fig. S2). In negative ion mode the predominant peak is a matrix cluster and a limited number of ions were observed that could interfere with true signal in the phospholipid range, with the 1% CMC solution yielding the most potential for interference (Fig. S2). We further characterized gelatin-inflated lungs for potential ion suppression effects, finding no additional suppression effect resulting from the addition of gelatin into the lung tissue (Fig. S3). Edge-to-edge maintenance of lung tissue structure is important for tracing inhaled drug deposition, visualizing molecular signatures of lung infection and the immune response, and mapping fine molecular changes in cellular composition.

### Gelatin inflated lungs yield segmentation matching manual annotation

**Figure 3:**
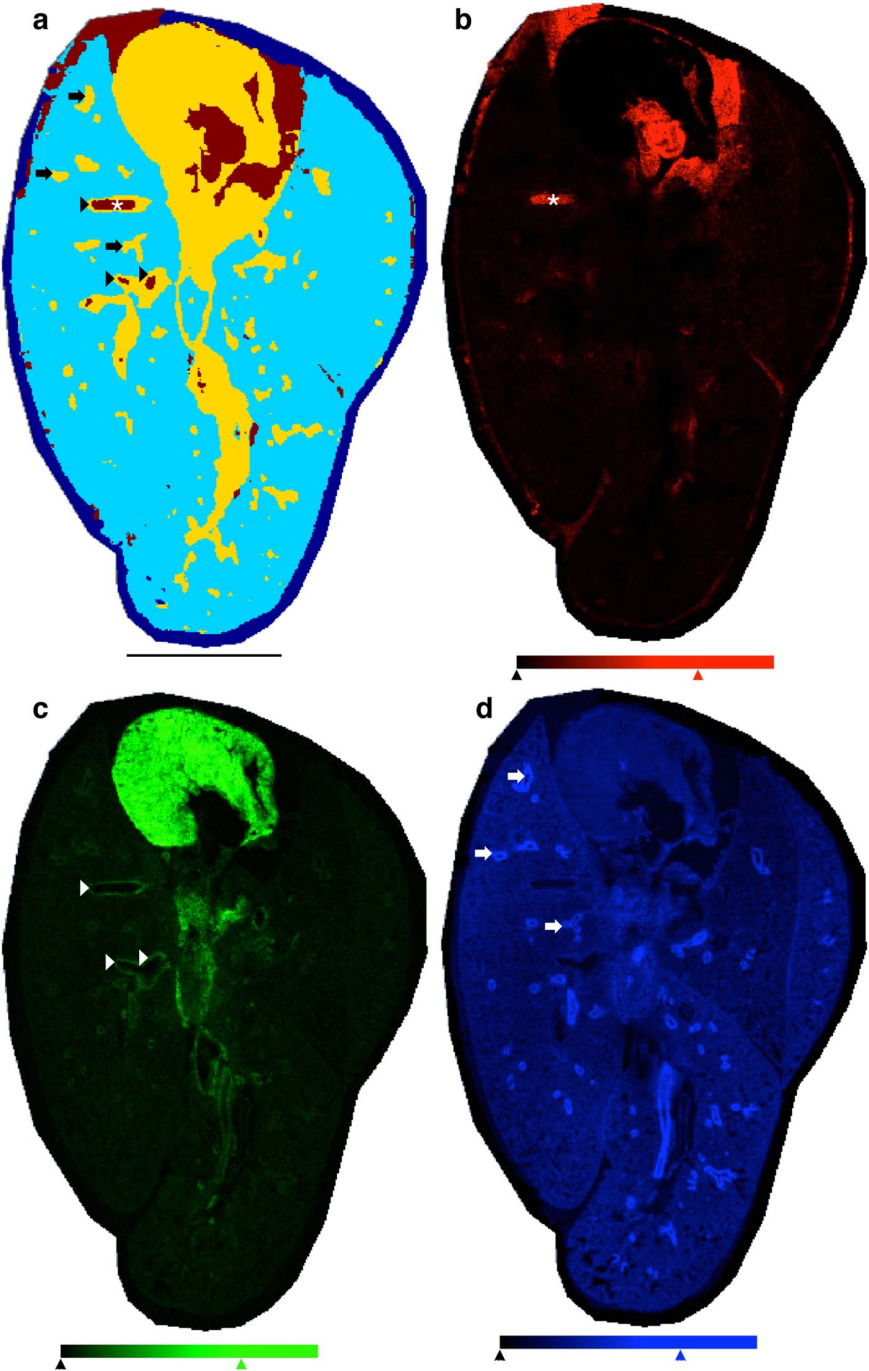
Three-component segmentation of inflated lungs discriminates blood, heart, and airway/vasculature structures matching gross histology and manual annotation. (**a**) Three-component segmented image (three-clusters, on-tissue) given in false color, dark blue component is off-tissue. Scale bar = 5 mm. Reference single channel images given for (**b**) blood - heme (*m/z* 615.1, red), (**c**) cardiac tissue - cardiolipin (*m/z* 1447.8, green), and (**d**) airway and parenchyma - PI 38:4 (*m/z* 885.5, blue). Annotations: asterisk - vessel containing blood, arrowheads - endothelial-lined vascular structures, arrows - epithelial-lined airway structures. MALDI-TOF, negative ion mode, 50 μm spatial resolution, normalized to TIC. (**b**–**d**) Single channel images: color reference bars with limiters as shown 0–70% relative intensity, all channels.

Given the diversity of the total area across the inflated lung tissue, we determined whether an accurate segmentation map could be generated from the imaging data to reflect a manual annotation of lung histology. Unsupervised hierarchical clustering of MSI data is a machine learning approach that classifies each spectrum into a cluster. The resulting dendrogram can be positionally mapped to tissue using whole cluster nodes based on the location of each underlying spectrum; in this manner it is a practical data reduction tool for interpretation of large and/or complex MSI datasets. From a gelatin inflated lung section, we generated a spatial segmentation map. The first three segmentation levels (excluding the component spectrum corresponding to the ITO surface surrounding the tissue) described the four major components present in the tissue section: the heart, blood, vascular, and airway linings separately from the lung parenchyma (Figs. 3a, S4). In the segmented image, the brick red areas (Fig. 3a) are composed of the spectra from 4881 pixels co-register to the areas of high heme intensity (*m/z* 615.1) (Fig. 3b), including a blood vessel in the lung tissue (asterisk) and blood-filled chambers of the heart. We observed similarly matching segmentation (yellow, average spectrum representing 17,027 pixels, Figs. 3a, S4) with regards to the cardiac tissue visualized by [CL(72:8)-H]^−^ (*m/z* 1447.8) (Fig. 3c). In addition to the [CL(72:8)-H]^−^ signal corresponding to the yellow segment (Fig. 3a), endothelial-lined vascular structures (arrowheads, Fig. 3c) were resolved along with the epithelial-lined airway structures (arrows, Fig. 3d) using the mass channel *m/z* 885.5 assigned [PI(38:4)-H]^−^ (Fig. 3d). The lung parenchyma (consisting of 51,561 pixels colored in cyan, Fig. 3a**)**, previously described by low-to-moderate intensity detection of the ion *m/z* 885.5 was also segmented from the higher abundance airway features.

**Figure 4:**
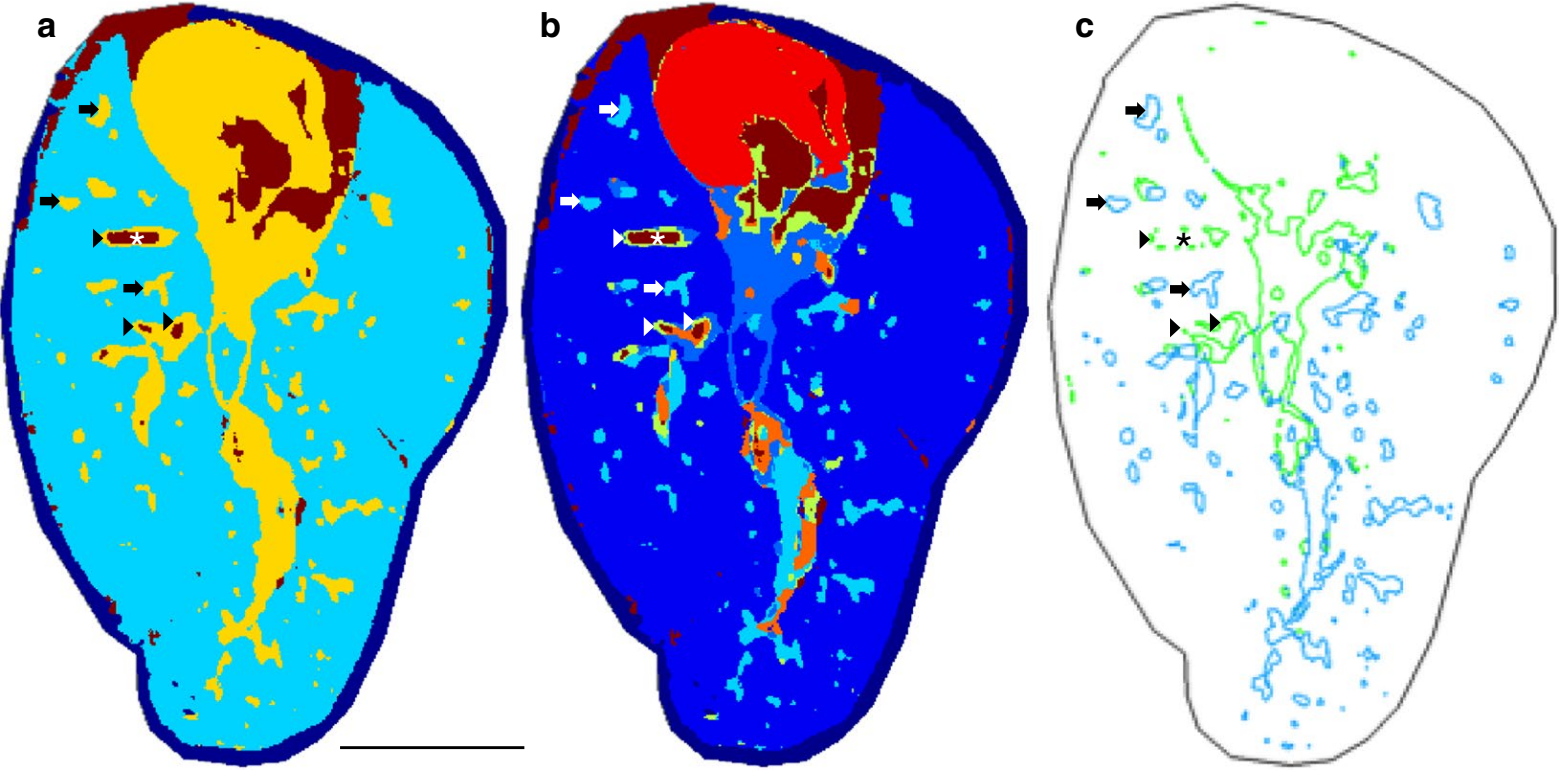
Unsupervised clustering is sufficient to discriminate endothelial vasculature from epithelial airway structures within nine segments. (**a**) Three-component segmented image (three clusters on-tissue) given in false color, dark blue component is off-tissue. (**b**) Nine-component segmented image (nine clusters on-tissue) given in false color, dark blue component is off-tissue. (**c**) Pixels defining the edges of the segment characterized by the average spectrum representing endothelial-lined vascular structures (lime) and the epithelial-lined airway structures (blue). Scale bar = 5 mm. Annotations: asterisk - vessel containing blood, arrowheads - endothelial-lined vascular structures, arrows - epithelial-lined airway structures. MALDI-TOF, negative ion mode, 50 μm spatial resolution, normalized to TIC.

We next determined if the combined yellow channel (Fig. 3a) representing cardiac, vascular, and airway structures could be further divided into spectral clusters defining each histological feature type independently. Upon further segmentation of the spectra clustering from these 17,027 yellow pixels (Figs. 3a, 4a and S4), we found separation of these distinct histological features six nodes (branches into the clustering dendrogram) away from the parent cluster (Figs. 4b, S4). In the refined segmentation map, the heart is represented by 6168 red pixels (Fig. 4b) defined by a common spectrum, which is separated from the vascular and airway components. The endothelial linings (and some areas of the blood/cardiac interface) are separated (1681 pixels, Figs. 4b, S4) and shown in lime green (arrowheads, Fig. 4b). Unsurprisingly, these areas line the brick red (4881 pixels, Figs. 4b, S4) components previously shown to have co-registered with areas characterized by a high intensity heme signature. The areas characterized as airway structures were lined with the ion *m/z* 885.5 segmented into 4124 pixels shown in light blue (arrows, Fig. 4b) resulting in sufficient separation in the component spectra to discriminate between endothelial and airway linings. Of the 57 of airway features described using ion *m/z* 885.5, all 57 were accurately identified by the segmentation shown in light blue (Figs. 4b, S5).

**Figure 5:**
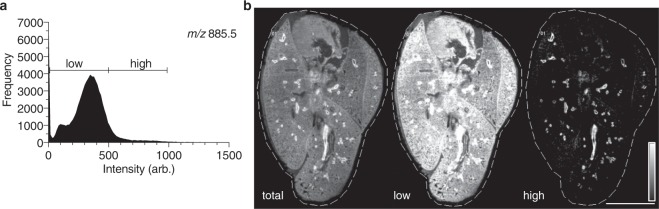
Discrimination of lung endothelial and airway structures is characterized by intensity of multiple PIs. (**a**) Frequency histogram of ion *m/z* 885.5, with low/high partition for (**b**). (**b**) Complete image for ion *m/z* 885.5 (intensities 0–1000, arbitrary), “all”-intensities. “Low”-intensity ion image for *m/z* 885.5 (0–500, arbitrary). “High”-intensity ion image for *m/z* 885.5. Color scale intensities (arb.) as given above for each channel. Scale bar = 5 mm.

To further characterize the spectral components driving the discrimination between the endothelial-lined vascular structures and the epithelial-lined airway structures, we interrogated the average spectrum of their respective segments. The pixels given in light blue (Fig. 4c) are represented by the spectrum of the same color (Fig. S6) and correspond to the epithelial-lined airway structures. Likewise, the pixels given in lime green (Fig. 4c) are represented by the inverted spectrum (Fig. S6a), corresponding to the endothelial-lined vascular structures. One of the driving components of the airway-associated spectrum is the high-intensity ion *m/z* 885.5 that is found in lower intensity in the vasculature-associated spectrum (Figs. 4c, S6d). In contrast, one of the major components of the spectrum representing the endothelial linings is *m/z* 1447.8 ([CL(72:8)-H]^−^), in addition to a lower abundance of high mass lipids (>*m/z* 1600, Fig. S6e) and decreased intensity of phospholipids (*m/z* 700–900) (Fig. S6d). We next divided the total *m/z* 885.5 ion window into two intensity profiles based on the frequency histogram (low and high, Fig. 5a) and plotted them separately to further investigate the segmentation results. The pixels with the highest abundance of *m/z* 885.5, though low frequency, were exclusively mapped to the major airway features whereas the low-to-moderate intensity pixels were present throughout the parenchyma as well as the airways (Fig. 5b). By preserving the histology of the lung, accurate segmentation mapping was possible using unsupervised hierarchical clustering. This is an important feature for future uses of the gelatin-inflated lung for automated bioinformatic mining of large-scale, comparative lung MSI studies.

### 3D Airway reconstruction possible with gelatin inflated lungs

To explore unique lung pathologies by MSI, rapid, fully intact 3D reconstructions of tissue features is necessary. Here, we reconstructed greater than 300 microns of serial tissue depth to evaluate our gelatin inflation method for high spatial resolution reassembly of unique lung structures. Consecutive serial sections (24) were taken near the center mass of the inflated lung tissue, representing a total tissue thickness of 312 μm (13 μm each). These sections were subsequently imaged in negative ion mode at 50 μm lateral resolution by high speed MALDI-TOF. All tissue sections in the series contained large and small airways, vascular features, heart, and blood components. 3D reconstructions were assembled from the three ions shown in Figs. 1 and 2. Consistent with the observations from single tissue sections heme reconstructed the blood-containing heart chambers and blood-filled large vessels in the lung (Fig. S7, video S1), cardiolipin reconstructed the heart tissue in the serial tissue sections (Fig. S8, video S2) and SAPI recreated the parenchyma through the full tissue thickness as well as large and small airways (Fig. S9, stills used for video; Fig. S10 video S3; Fig. S11 mass windows). All structural features were readily reconstructed in 3D, including several small airways observed on the right side of the lung that increased in diameter through the tissue. At 50 μm spatial resolution, the lung inflation method presented here results in high quality serial sections capable of producing highly detailed 3D reconstructions.

### High spatial resolution imaging and lipid identification

**Figure 6:**
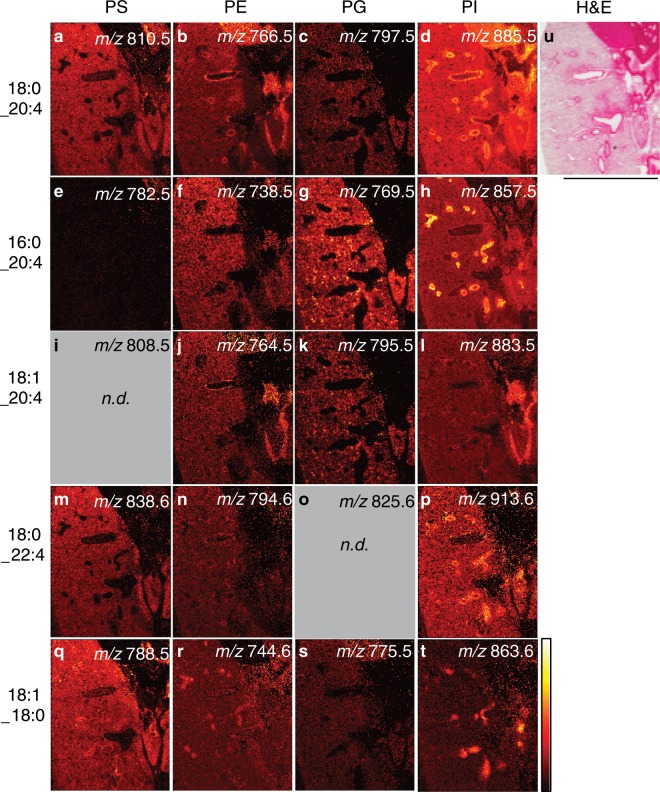
Arachidonic acid-containing PIs in the airway show biased toward a fully saturated acyl chain partner. (**a**–**t**) Parallelized lipidome image of a subset of inflated mouse lung, negative ion mode, DDA-image of alternating pixels. Phospholipid headgroups: phosphatidylserine (PS), phosphatidylethanolamine (PE), phosphatidylglycerol (PG), PI, with assigned primary identities (carbon#:unsaturation#, *sn-*positioning not assigned). Serial section of Fig. 1. Color bar = 0–100% relative intensity. Scale bar = 5 mm. 15 μm rastering with 30 μm final pixel rendering of MS1 image. Channels as given, ±0.005 Da. *n.d*. = not detected. (**u**) H&E stain, post-MSI.

Using the DDA-imaging method, we identified the specific lipid species localized to the airways. In particular, we interrogated the distribution of PUFA-containing phospholipids, specifically those containing arachidonic acid (20:4) fatty acyl chains. We have previously shown that PUFA-PIs, in particular SAPI, serve as pools for lethal lipid-based inflammation during infection with *Francisella novicida*^27^; therefore, we sought to localize these and other PUFA-containing determine their localization within in the lung. SAPI has previously been associated with airways in lung samples from mouse and human^25,35^. Based on this analysis, we were able to identify specific 20:4-containing phospholipid species that are enriched in the airway regions (Fig. 6). Accurate *m/z* values and MS/MS fragments used to support identifications presence and are provided in Table S1. Note the lipid identification identities presented here are based on the dominant contributing fragments that are consistent with the sum-composition assignment and in some cases isomeric species varying in individual fatty acyl compositions are also observed. Based on previous reports^25^, *m/z* 857.5 (Fig. 6h) and 885.5 (Fig. 6d), corresponding to [PI(16:0_20:4)-H]^−^ and [PI(18:0_20:4)-H]^−^, respectively, were predominantly distributed in the large airway epithelium (Fig. 6h,d). These two ions were the most abundant in the summated spectrum (see Fig. S6b) and were driving components of the spatial segmentation separating airway epithelium versus vascular endothelium.

Other lower abundant PUFA-containing PIs were also observed preferentially, within airway epithelium, specifically the C22:4-containing [PI(18:0_22:4)-H]^−^ (Fig. 6p). Interestingly, and in contrast to the airway-biased intensity of [PI(18:0_20:4)-H]^−^, the distribution of the [PI(18:1_20:4)-H]^−^ is absent from the airway (Fig. 6l). However, PIs containing 18:1 fatty acyls are not entirely absent from the airway, as an ion *m/z* 863.6 (Fig. 6t, [PI(18:0_18:1)-H]^−^) is observed in the airways at low abundance. Since we found that the distribution of [PI(18:0_20:4)-H]^−^ and [PI(18:1_20:4)-H]^−^ were different, we interrogated the matching acyl compositions of PG. With respect to the 18:0_20:4 acyl configuration versus the 18:1_20:4 configuration, PG lipids were observed with matching distribution patterns with [PG(18:1_20:4)-H]^−^ (Fig. 6k) present in high intensity puncta throughout the parenchyma and [PG(18:0_20:4)-H]^−^ (Fig. 6c) found to be co-localized, though at lower intensity. It is possible that spatial separation of AA-containing PI lipids based on saturation state of C18:0 and is a headgroup-limited phenomenon and other classes such as (phosphatidylcholine) PC should be investigated. The 16:0-based PG ion [PG(16:0_20:4)-H]^−^ (Fig. 6g) also co-localized with [PG(18:0_20:4)-H]^−^ and [PG(18:1_20:4)-H]^−^. The pixels containing PG described as ‘punctate’ were investigated further to ensure that co-localization was on tissue, not in larger gelatin inflated alveolar areas. Limited to the lung tissue (not the nearby cardiac tissue), we found that [PE(18:0_20:4)-H]^−^ observed at *m/z* 766.5385 was present in modest abundance in the airways (Fig. 6b). Our results demonstrate that the predominant pool of AA-containing phospholipids detected in the negative ion mode in the airway lining are biased to the PI headgroup, whereas other AA-containing classes (PS, PE, PG) are only marginally detected and colocalized to the airway lining. While these studies focused on the negative mode PUFA-containing PLs, we^7,36^ (and others^35^) have demonstrated abundant PUFA-PC in the airways. These results underline the tight control of structurally-specific phospholipid pools within discrete cell populations, further highlighting the need for advances in single-cell resolution MSI for routine application^15,16^.

## Discussion

In this work we present a lung inflation method free from excessive exogenous compounds and compatible with advanced lipidomic imaging, volume reconstruction, and unsupervised spatial segmentation. Thin sectioning and mounting fresh frozen tissues is a nominal task with solid organs, such as kidney, spleen, liver, and brain; however, working with lung tissue by the same methods is challenging due to the open, lacey nature of the alveoli. For this reason, lung tissue must be inflated to elucidate relevant airway structures and for accurate interpretation of molecular histology data. Lipid and metabolite MSI studies are best performed with fresh frozen tissue preparations. Using mOCT, a balanced salt solution and polymer mixture lacking benzalkonium preservative, illustrated the importance of lung inflation for MSI studies^25^. Another method for inflating rodent lungs for MSI was recently reported using a combination of phosphate buffered saline perfusion followed by inflation with a 1% CMC solution^26^. Using the perfusion/CMC inflation approach, gapped serial sections were collected for 3D reconstruction of high-density MALDI-FTICR MSI data, ultimately spanning the full tissue thickness. In addition to rodent lung inflation preparations, others have reported the importance of *post-hoc* inflation-fixation of nonhuman primate lung samples to obtain interpretable lipid MSI results^37^. Here, we developed an MSI-compatible inflation solution that contained as few exogenous components as possible, forgoing perfusion, fixation, and addition of salt solutions upon inflation.

Using a solution of 2% porcine gelatin in molecular grade water, we inflated mouse lungs in preparation for MSI allowing for *en bloc* removal of the heart and lungs. The advantage of this method is the introduction of porcine gelatin as the only exogenous component, an established, MSI-compatible tissue-mounting tool that does not interfere with downstream applications such as histological stains or immunostains. By this method, we prepared high quality, fully intact serial sections with morphology preserved from edge-to-edge within the center of the tissue for MSI data analysis of lung lipids.

In preparation for future studies comparing experimental conditions in lung, it was apparent that automated histological interpretation would be a necessary tool. Thus, we used unsupervised hierarchical clustering of the mass spectral data to first define major histological features within inflated lung and validated the accuracy using a traditional histology method (H&E). We found that gross anatomy of the whole section could be discriminated by three components: lung parenchyma, airway and vasculature features, and cardiac tissue. Further, we found that fine histological features, specifically the vasculature and airway, could be discriminated by nine total components. Accurate automated segmentation analysis of control tissues, such as demonstrated here is the precursor for comparative studies of lung pathologies by MSI because spectral comparisons of histological features for unique biomarkers can be done empirically. Our experiments were performed at 50 μm spatial resolution and therefore cannot address questions at the single cell level within the context of the whole lung; advances in high spatial resolution MSI for routine application will be necessary.

The component spectrum of the airway segment contained high abundance ions corresponding to multiple PUFA-PIs, including [PI(36:4)-H]^−^, [PI(38:4)-H]^−^, and [PI(40:4)-H]^−^ lipid species (Figs. 4,6). These findings agree with previous reports of high concentration arachidonic acid-containing anionic lipids in the airways of lung tissue. Our results largely concur with previous reports of distribution bias of PUFA-containing phospholipids to the airway epithelium^25,35^. The discordant mapping of [PI(18:0_20:4)-H]^−^ and [PI(18:1_20:4)-H]^−^ in this study suggests a tight control of the structure-specific AA-containing PI pool. This observation raises questions about the role of the phospholipid pool in regulating the structure-activity relationship between AA-containing PIs and the specificity of phospholipases and acyltransferases.

Our PUFA-PI findings in gelatin-inflated lungs illuminate a poor understanding of the underlying control mechanisms for the spatial distribution bias resulting from the length and complexity of the partnering acyl position in an arachidonate-containing PI at a whole tissue level. Arachidonate-containing phospholipids, especially the high abundance [PI(18:0_20:4)-H]^−^, were shown to contribute to rapid lethality in a model of *Francisella* infection due to the rapid induction of downstream pro-inflammatory metabolic products of free arachidonate through the cyclooxygenase-2 pathway^27^. Since the airways serve as a primary site of interaction with the environment, including host-pathogen interactions, it follows logically that these pools of PUFA-containing phospholipids are concentrated at the airway interface to serve as an early inflammatory response. Future studies of the relative changes in abundance of these arachidonate-containing phospholipids in the airway following various inflammatory insults will be important in understanding the control of downstream lipid mediators in spatially and temporally balancing pathological lipid inflammation (ex: PGE_2_) with the production of protective lipid mediators (ex: resolvins)^38^. PUFA-PCs, –PEs and –PIs are a major source of cytosolic phospholipase 2 (cPLA2)-derived free AA upstream of eicosanoid production^39^. Previous studies have demonstrated that the enzymatic requirements of cPLA2 are influenced by anionic phospholipids, especially PI-bisphosphates^40,41^. The presence of PUFA-PIs at the airway lining with these and other PUFA-PLs implies regulated coordination of the cPLA2 substrate pool beyond our current understanding. Choline regulation in activated macrophages, in part through *de novo* production of PCs, is an important link between immunometabolism and the regulation of the total phospholipid pool during inflammatory insult at sites such as the airway^42^. Together, these results support the hypothesis that the ready-state pool of pre-inflammatory phospholipid substrates is tightly controlled upstream of the enzymes involved in lipid inflammation in order to match different phospholipase substrate preferences. Further, arachidonate recycling via the Lands^43^ pathway could be explored using this approach to determine whether there is coordinate control of the phospholipid pool alongside lysophospholipid reacylation^44^.

## Conclusions

In this work, we present a method for rodent lung inflation that introduces minimal exogenous material to the sample, preserves lung histology, is compatible with advances MSI techniques including parallelized inline spatial lipidomics and complete 3D reconstructions, and appropriate for unsupervised spatial interpretation. This advance in tissue preparation enables an entire field of molecular histology studies of non-traditional targets – especially lipids and metabolites – in models of lung pathology ranging from environmental insults, genetic functional perturbations and cancers to bacterial and viral infections.

## Supplementary information

Video S1:3D rendering of heme in lung from Figure S7

Video S2: 3D rendering of cardiolipin in lung from Figure S8

Video S3: 3D rendering of SAPI in lung from Figure S10

Supplemental Information Package

## Data Availability

The data included in this manuscript are available upon reasonable request from the corresponding author. Instructional materials for the inflation method will be available upon request.
