# Supplementary figures and images for: Maintenance of Deep Lung Architecture and Automated Airway Segmentation for 3D Mass Spectrometry Imaging
Source: Sci Rep. 2019 Dec 27;9:20160. doi: 10.1038/s41598-019-56364-4 (PMC6934789; doi:10.1038/s41598-019-56364-4)

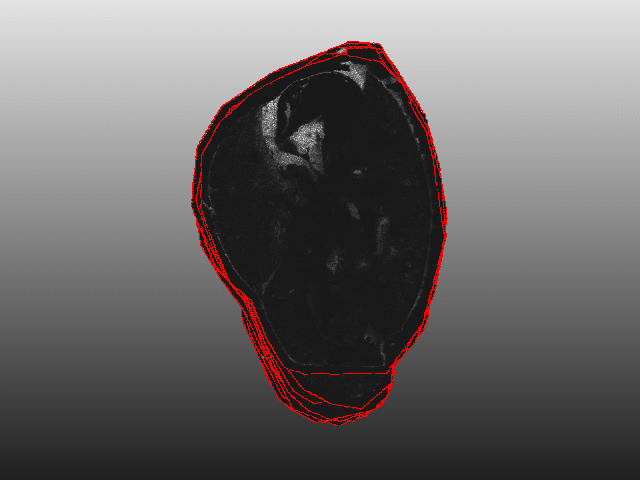

Supplement: Supplementary file 1 — Video S1:3D rendering of heme in lung from Figure S7 [file 41598_2019_56364_MOESM1_ESM.gif]

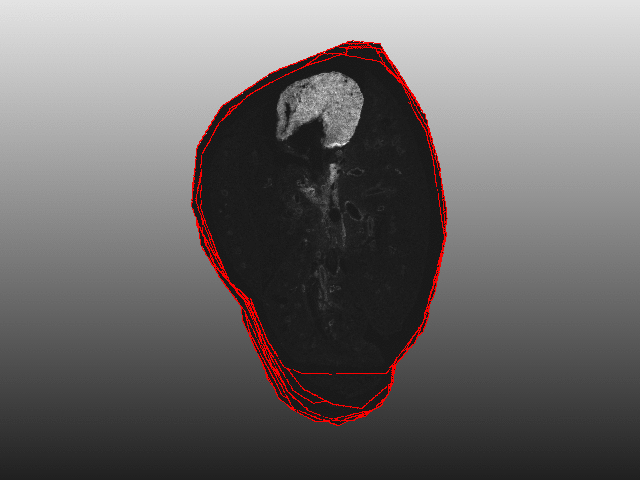

Supplement: Supplementary file 2 — Video S2: 3D rendering of cardiolipin in lung from Figure S8 [file 41598_2019_56364_MOESM2_ESM.gif]

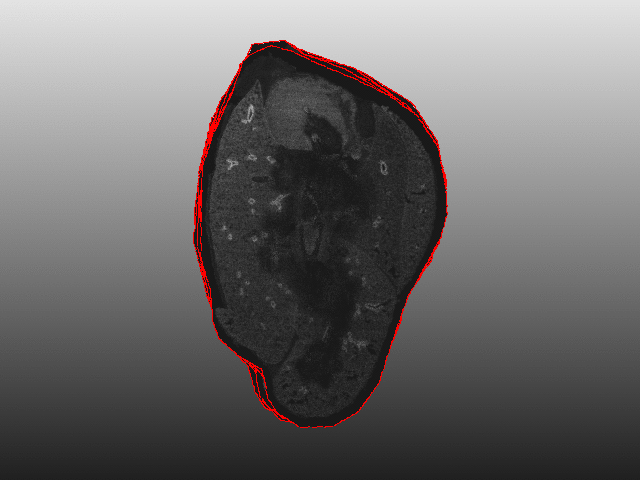

Supplement: Supplementary file 3 — Video S3: 3D rendering of SAPI in lung from Figure S10 [file 41598_2019_56364_MOESM3_ESM.gif]
