# Supplementary material for: Maintenance of Deep Lung Architecture and Automated Airway Segmentation for 3D Mass Spectrometry Imaging
Source: Sci Rep. 2019 Dec 27;9:20160. doi: 10.1038/s41598-019-56364-4 (PMC6934789; doi:10.1038/s41598-019-56364-4)
Supplement: Supplementary file 4 — Supplemental Information Package [file 41598_2019_56364_MOESM4_ESM.pdf]

SUPPLEMENTAL INFORMATION FOR:

Maintenance of Deep Lung Architecture and Automated Airway Segmentation for 3D Mass Spectrometry Imaging

Alison J. Scott[1,2], Courtney E. Chandler[1], Shane R. Ellis[2], Ron M.A. Heeren[2], Robert K. Ernst[1]

1) Department of Microbial Pathogenesis, University of Maryland School of Dentistry, MD 21201

2) Maastricht Multimodal Molecular Imaging (M4I) Institute, Maastricht University, NL 6229ER

Corresponding Author:

Robert K. Ernst

Department of Microbial Pathogenesis, School of Dentistry

University of Maryland, Baltimore

650 W. Baltimore St

Baltimore, MD 21201

rkernst@umaryland.edu

1 410 706 3622

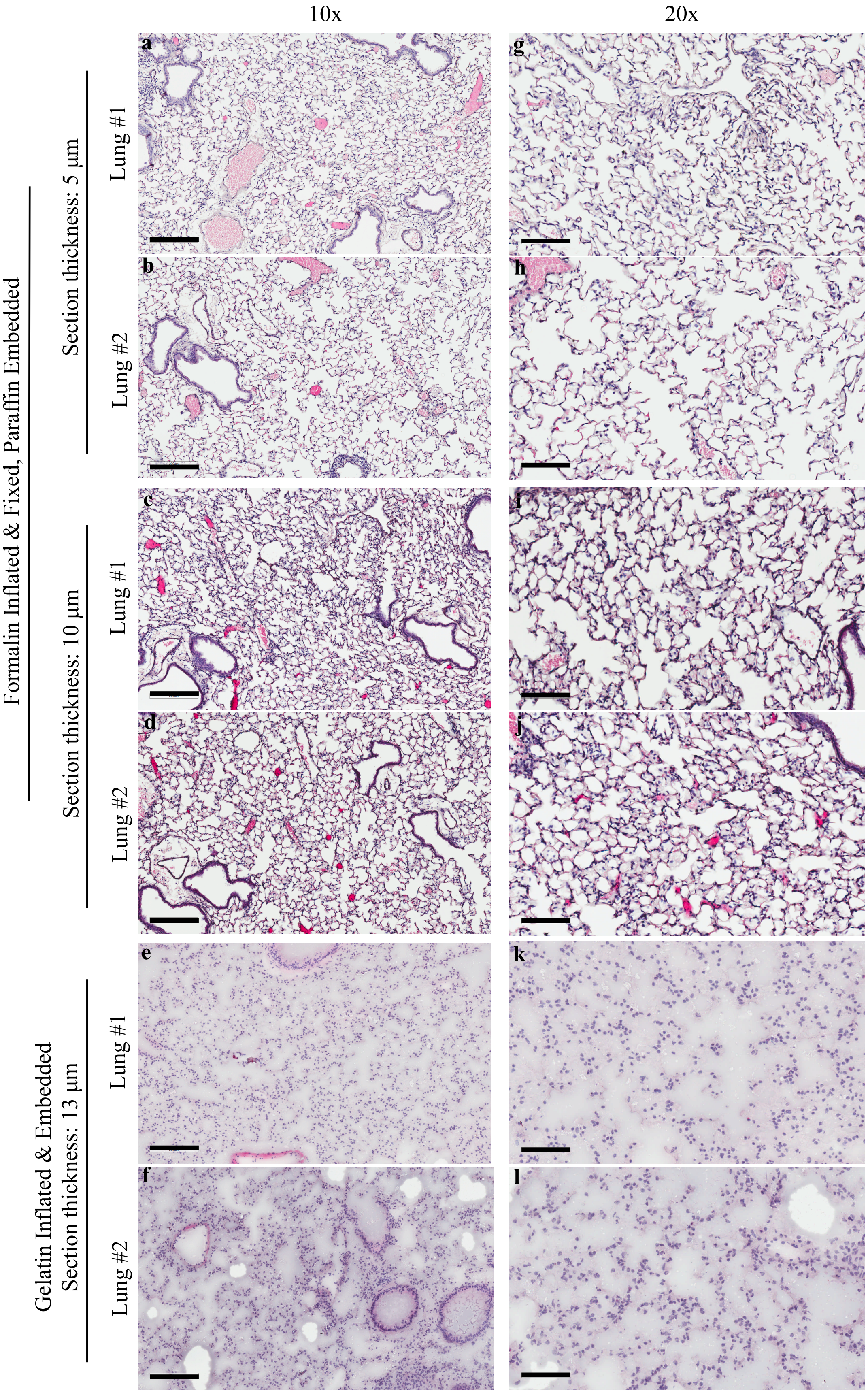
10x
20x
a
b
c
d
e
f
g
h
i
j
k
l
Formalin Inflated & Fixed, Paraffin Embedded
Section thickness: 5 µm
Section thickness: 10 µm
Gelatin Inflated & Embedded
Section thickness: 13 µm
Lung #1
Lung #2

Figure S1: Gelatin inflated lung histology resembles that of traditionally prepared FFPE samples. Two mouse lungs were inflated: 1) using neutral buffered formalin followed by formalin fixation and paraffin embedding (a-d, g-j) or 2) per this protocol using gelatin and cryopreparation (e, f, k, l). FFPE-prepared lungs were sectioned at the standard thickness (5 µm; a, b, g, h) or double thick (10 µm c, d, i, j) and stained by H&E. Gelatin prepared lungs were sectioned at the thickness described in this protocol (13 µm) and stained by H&E without MSI analysis. Two magnifications are shown: 10x (a-f, scale bar = 200 µm) and 20x (g-l, scale bar = 100 µm).

Inflation mixtures lack interfering signal in lipid range

The popular embedding medium optimal cutting temperature (OCT) is commonly avoided[1] for MSI experiments for a variety of reasons and alternative embedding solutions are found throughout the literature. We sought to evaluate the relative interference from reported sample embedding mixtures in the phospholipid mass range in both positive and negative modes. It is worth noting that for every unique experimental application (metabolite imaging, peptide and protein imaging, etc.) these parameters should be re-evaluated for suitability and optimal performance. In addition to the 2% gelatin solution (in water) reported here, we evaluated 50% OCT in PBS[2], 50% OCT in water, and 1% CMC in water[3]. To best represent the quantity of embedding mixture present in inflated lung tissue we prepared frozen plugs of each embedding/inflation solution and sectioned them at the same thickness as the lung tissues. These plug sections were then prepared with NRM matrix and analyzed in both positive and negative ion modes on a MALDI-TOF MS. The major ions detected in negative mode were predominantly matrix monomer and associated clusters, with 1x NRM as the base peak in all spectra: negative mode - *m/z* 167.1 and positive mode – *m/z* 169.1. Representative spectra (averaged of 3) are presented in **Fig. S2**. The resulting positive mode spectra in the phospholipid mass range resembled noise spectra in each condition. The resulting negative mode spectrum for each condition showed a common NRM matrix cluster and the OCT conditions and gelatin condition had the fewest ions that might interfere in the phospholipid range.

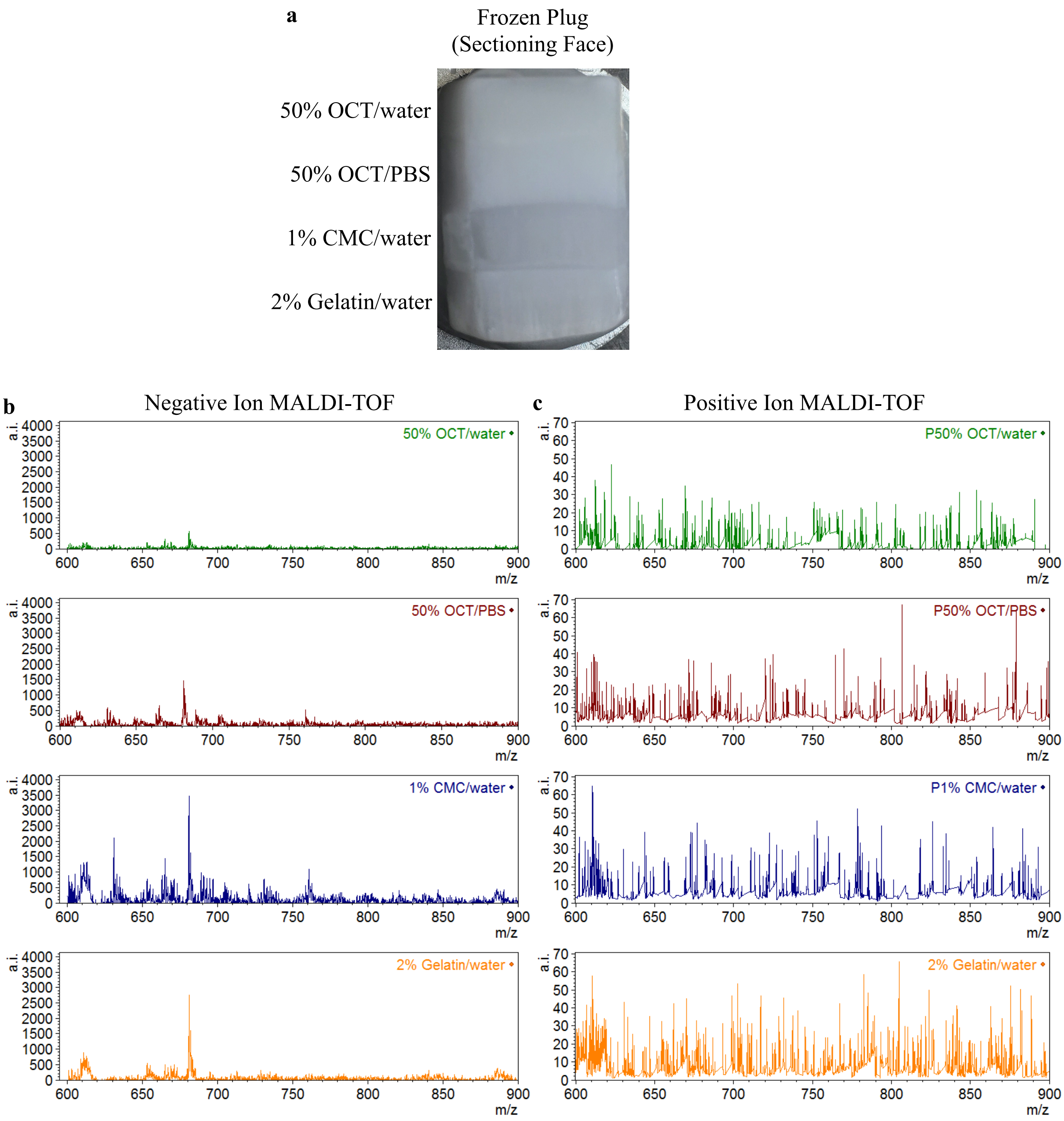

Figure S2: Comparison of baseline spectrum in the phospholipid range from various embedding solutions prepared with matrix. Spectra were collected (*m/z* 100-1000) in both positive and negative ion modes on a MALDI-TOF from triplicate preparations of thinly sectioned embedding matrices (a). Phospholipid range (*m/z* 600-900) is presented (b-c). Average spectra from triplicate samples, arbitrary intensity (a.i.), calibrated to NRM clusters.

Inflation Does Not Contribute to Ion Suppression

Ion suppression is a common obstacle in MSI studies; therefore, we sought to evaluate that the inflation method using 2% gelatin did not aggravate suppressive effects inherent to the tissue itself. Thin sections of 2% gelatin alone, air inflated mouse lungs, and 2% gelatin inflated mouse lungs were mounted onto ITO glass slides and used as the underlying substrate for detection of a standard, exogenous phospholipid spotted in serial dilution. Since phosphatidylcholines (PCs) are the most abundant class of phospholipid in mammalian lung[4], we characterized ion suppression based on PC detection in positive mode. We used a synthetic PC 31:1 (PC 17:0/14:1) to evaluate ion abundance. PC 17:0/14:1 was detected as a positive ion *m/z* 718.5381 $[M+H]^+$ by MALDI-FTICR in a spot-profiling experiment. We tested a four-log dilution series (14 nM, 140 nM, 1400 nM, 14000 nM) of PC 17:0/14:1 spotted on inflated lung tissue or control substrates to establish a detection range. Notably, $[PC(17:0/14:1)+H]^+$ was neither detected in lung tissue sections (air or gelatin inflated) nor in the gelatin alone control sections. Spotted directly onto ITO glass $[PC(17:0/14:1)+H]^+$ was detected at 140 nM and showed a concentration-dependent intensity response increasing through 14000 nM (**Fig. S3**). Detection results were similar when the standard was spotted onto a section of a 2% gelatin plug with the lower detection for $[PC(17:0/14:1)+H]^+$ at 140 nM and a concentration-dependent increase observed through 14000 nM (**Fig. S3**). The lowest detectable concentration of the control lipid on sections of air-inflated lungs was the maximum tested, 14000 nM, with no detectable signal at lower concentrations (**Fig. S3**). In contrast, gelatin inflation of the lung improved detection of the exogenous lipid over air-inflated lungs with the lowest concentration detected at 1400 nM (**Fig. S3**), though compared to the gelatin plug the presence of lung tissue conferred a log lower detection. These results confirm that gelatin inflation of lung is an improvement over unprepared

lung tissue (air inflated) and at least two logs of working range can be achieved for exogenous standards.

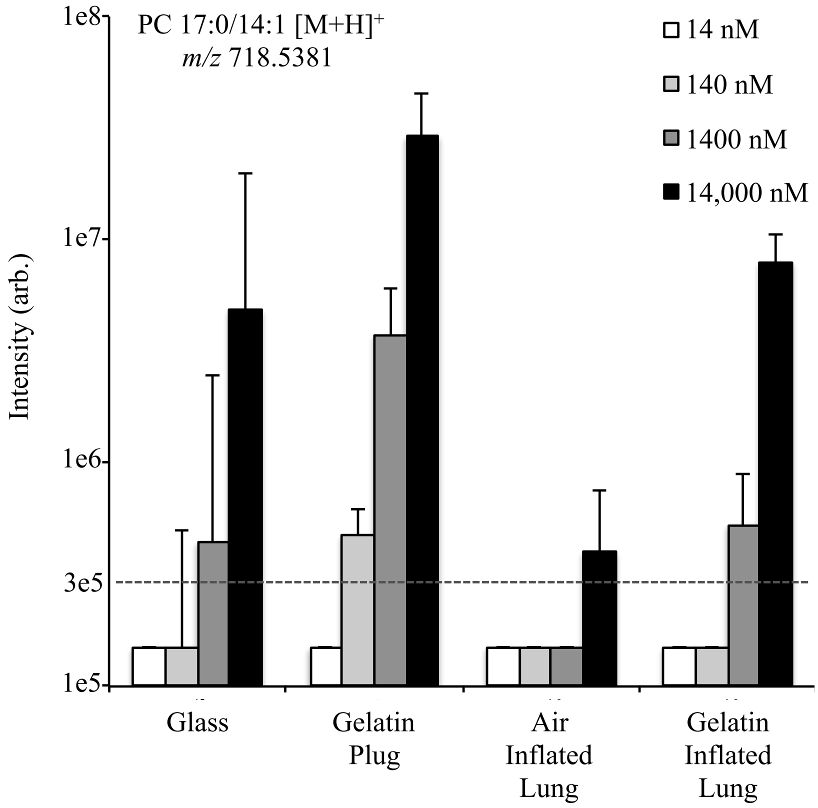

Figure S3: Gelatin inflation of the lung improves detection of exogenous, spotted lipid compared to untreated lung tissue. Arbitrary intensity of PC 17:0/14:1 detected as the $[M+H]^+$ ion *m/z* 718.5381 +/- 3ppm on untreated ITO glass, a gelatin plug section, air inflated mouse lung section, and gelatin inflated mouse lung section (all 13 µm thickness). Spot profiling, MALDI-FTICR, positive ion mode, 5 positions per spot, average intensity +/- standard deviation using a minimum detection threshold of 300,000 intensity (arb.), ion not detected reported as 50% of threshold. Data representative of 2 independent experiments.

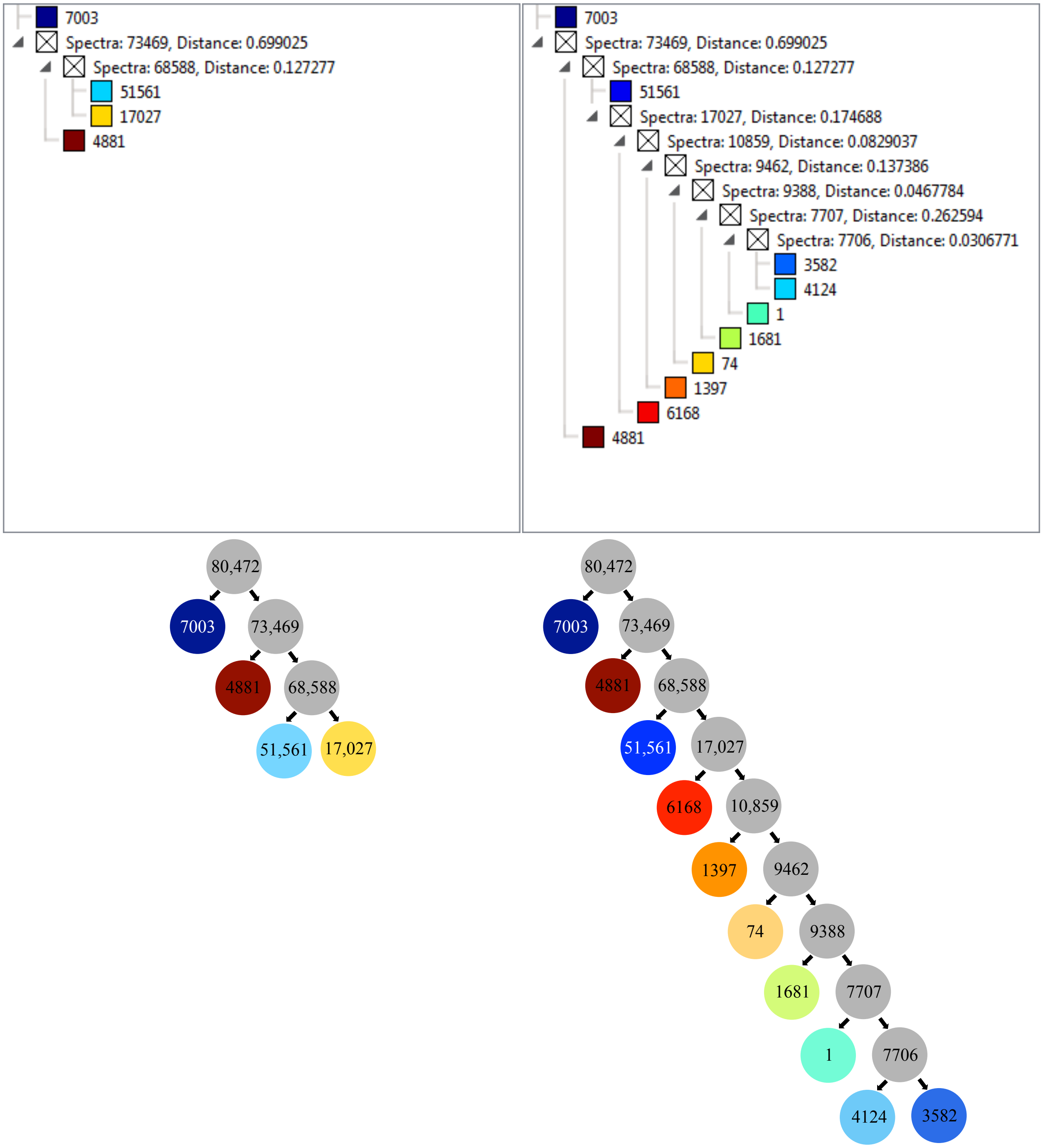

Figure S4: Hierarchical clustering of negative ion mode phospholipid imaging data. Clusters and false color scheme for **Figs. 3, 4** showing nodes, total spectra per node, and distances. (Left) Reference for three-component segmented image. (Right) Reference for nine-component segmented image. (Both) Dark blue node (7003 spectra) is off-tissue. Cartoon dendrograms given in the bottom panel were reproduced from clustering result in top row.

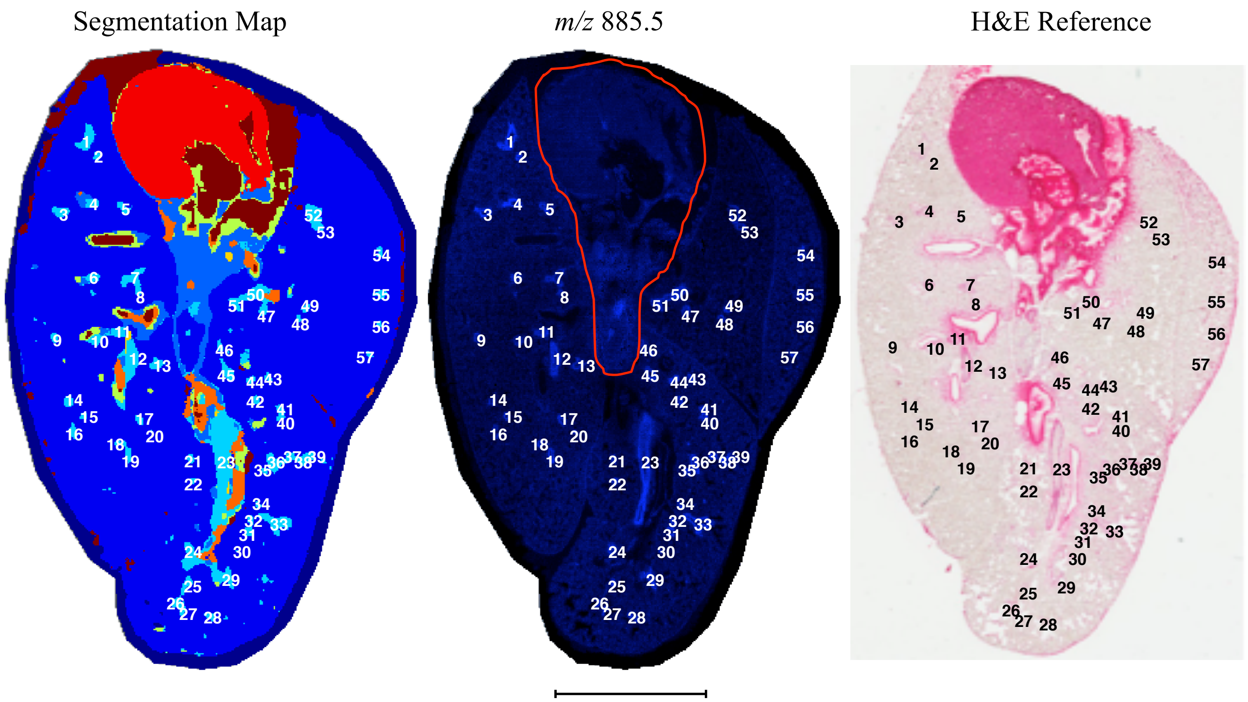

Figure S5: Feature count comparing airway assignments by single ion image to histology and nine-component segmentation. (Left) Nine-component segmented image (nine clusters on-tissue) given in false color from **Fig. 4b**, dark blue component is off-tissue. All annotated features from single ion channel map correspond to blue segment. (Middle) Single ion channel map of *m/z* 885.5 with high intensity airway features enumerated. Image and intensity from **Fig. 3**. (Right) H&E reference image for histology. All annotated features from single ion channel map correspond to airway features. Scale bar = 5 mm. Red outlined area not evaluated (area contains cardiac tissue, blood, and other non-lung tissues).

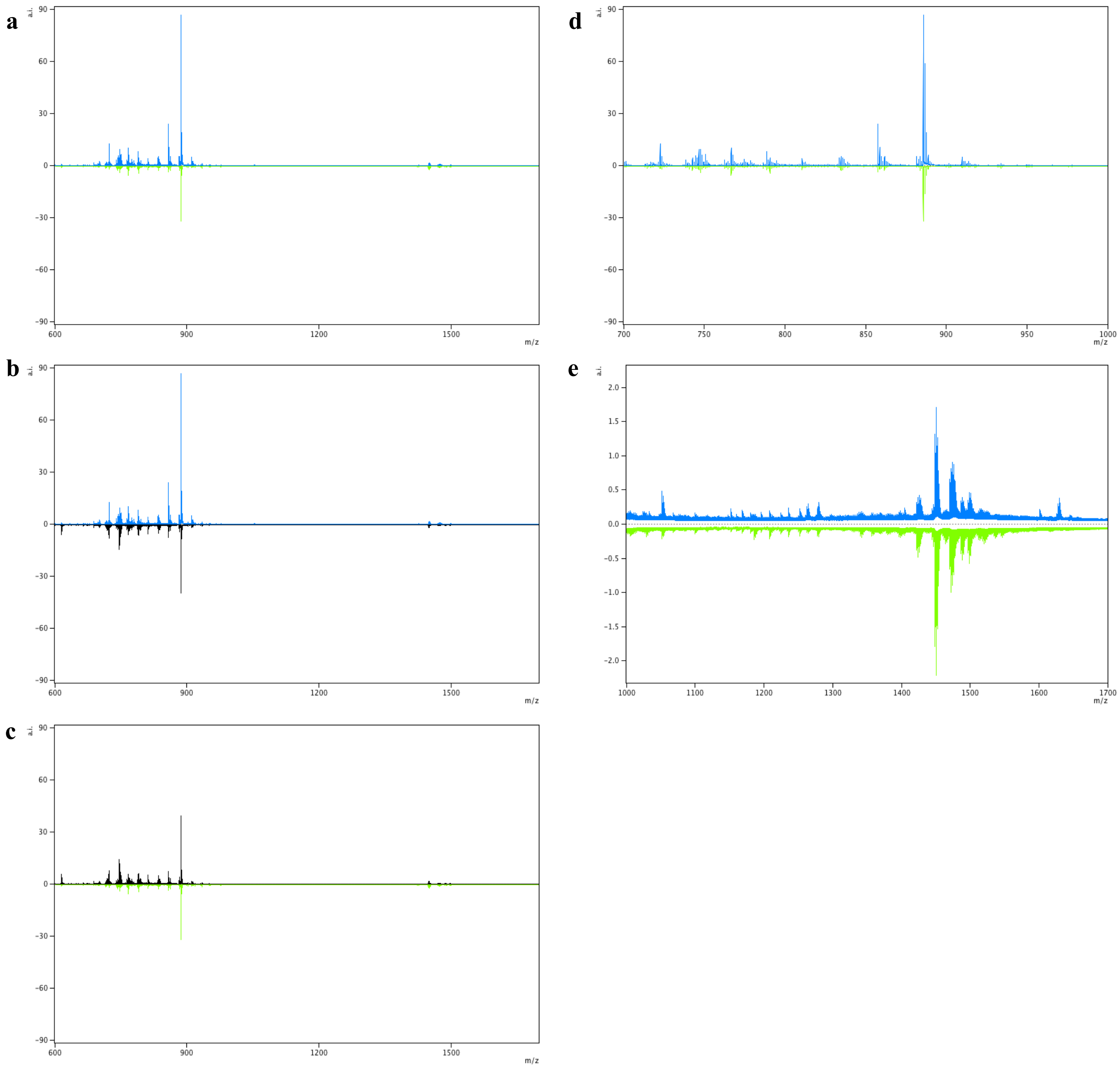

Figure S6: Spectral aspects of total lung image, airway, and vascular features. (a-c) Average spectra from total lung image (black), airway segment (blue), and vascular segment (lime) from full mass range collected. Same y-axis for direct comparison. (d-e) Spectral window zoomed to areas of interest; y-axes as given. MALDI-TOF, 50 μm spatial resolution, negative ion mode, normalized to TIC, then extracted for spectral display.

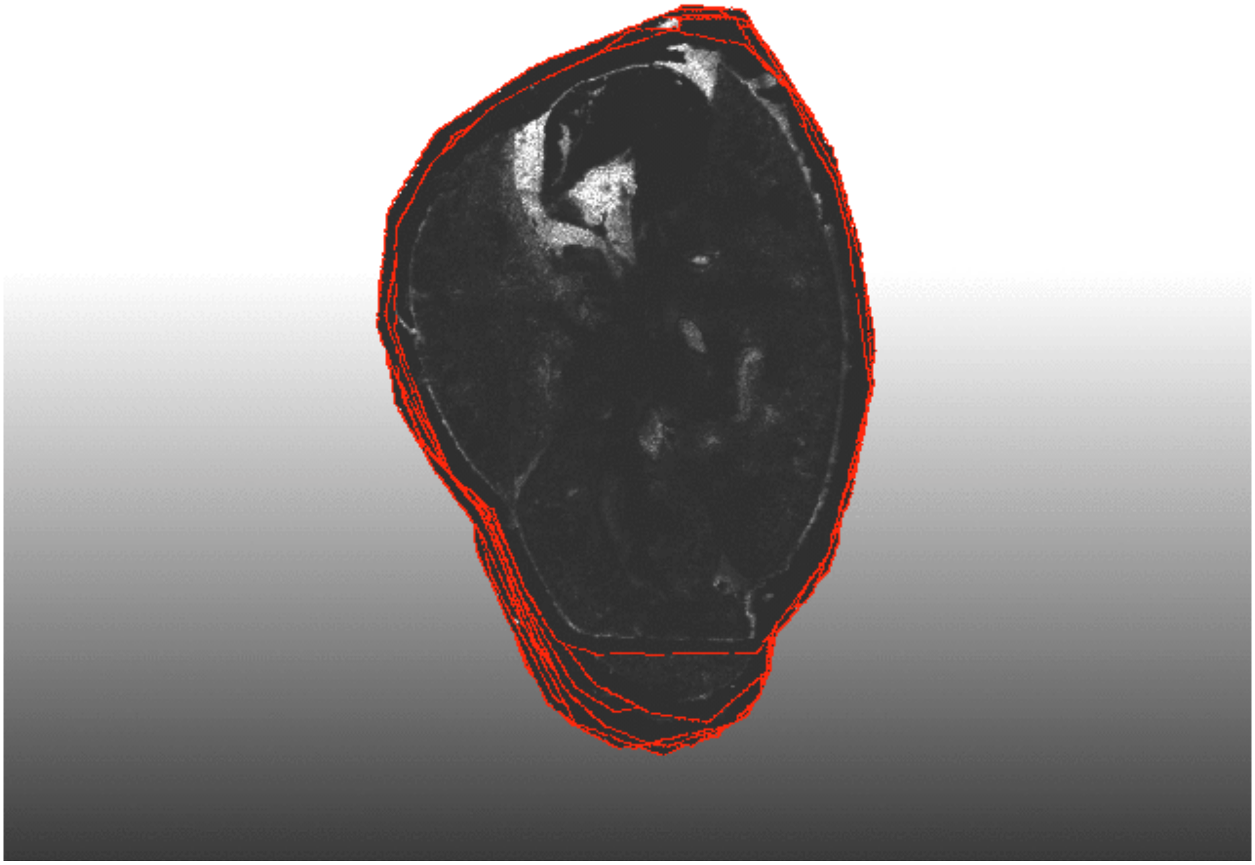

Figure S7: 3D video rendering of *m/z* 615.1 from 24 consecutive lung sections. Z-stack reconstruction of 24, 13 µm sections cut from the center mass of the gelatin inflated mouse lung tissue. MALDI-TOF, 50 µm spatial resolution, normalized to TIC, 360° rotation with both horizontal and vertical toggling, 30 seconds. Spectral reference and mass channel given in **Fig. S11**.

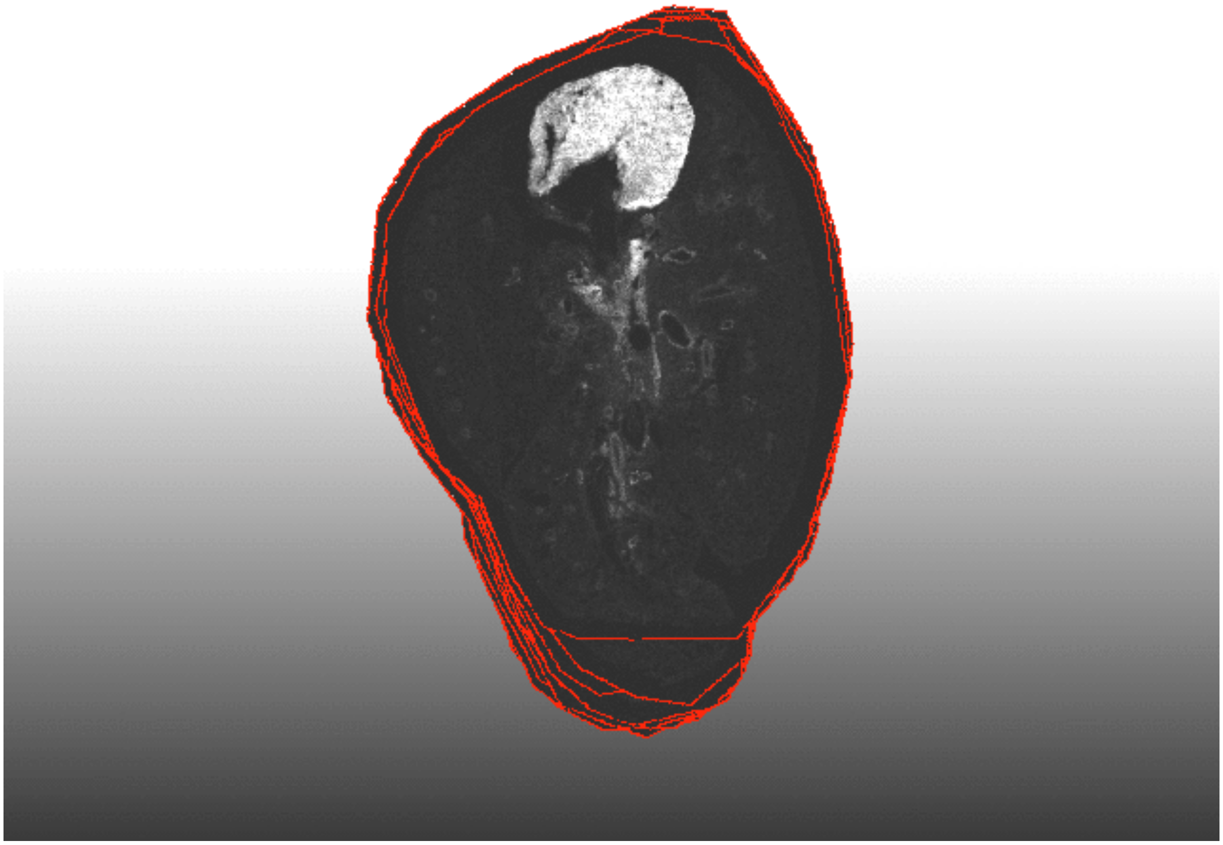

Figure S8: 3D video rendering of *m/z* 1447.8 from 24 consecutive lung sections. *Z*-stack reconstruction of 24, 13 µm sections cut from the center mass of the gelatin inflated mouse lung tissue. MALDI-TOF, 50 µm spatial resolution, normalized to TIC, 360° rotation with both horizontal and vertical toggling, 30 seconds. Spectral reference and mass channel given in **Fig. S11**.

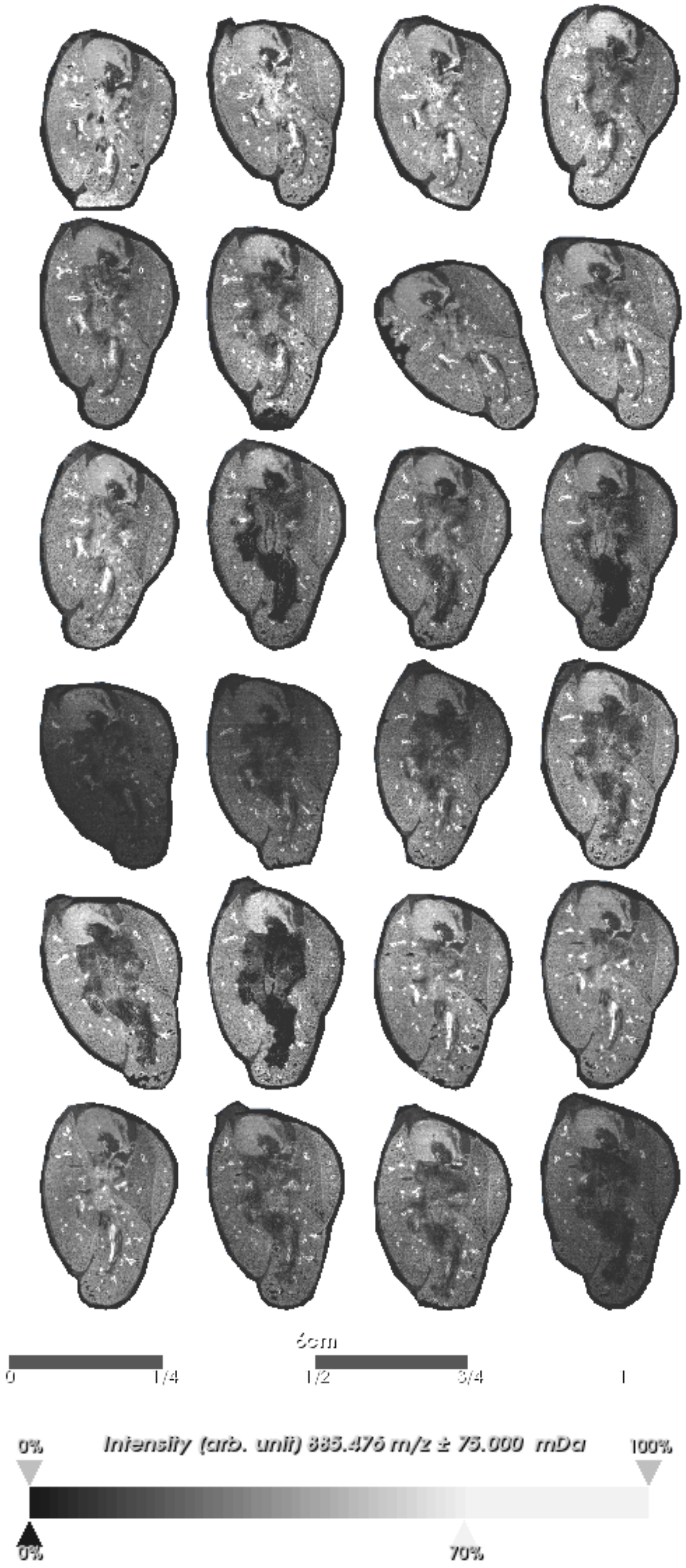
6cm
0
1/4
1/2
3/4
1
0%
Intensity (arb. unit) 885.476 m/z ± 75.000 mDa
100%
0%
70%

Figure S9: 2D images underlying 3D reconstruction video of *m/z* 885.5. Ion channel *m/z* 885.5 ± 75 mDa (corresponding to $[PI (38:4)-H]^-$) shown as 2D section stills. Scale and intensity bars as given for 2D image and 3D reconstruction video. MALDI-TOF, 50 µm spatial resolution, negative ion mode, normalized to TIC, intensity display optimized for print and video reconstruction accuracy.

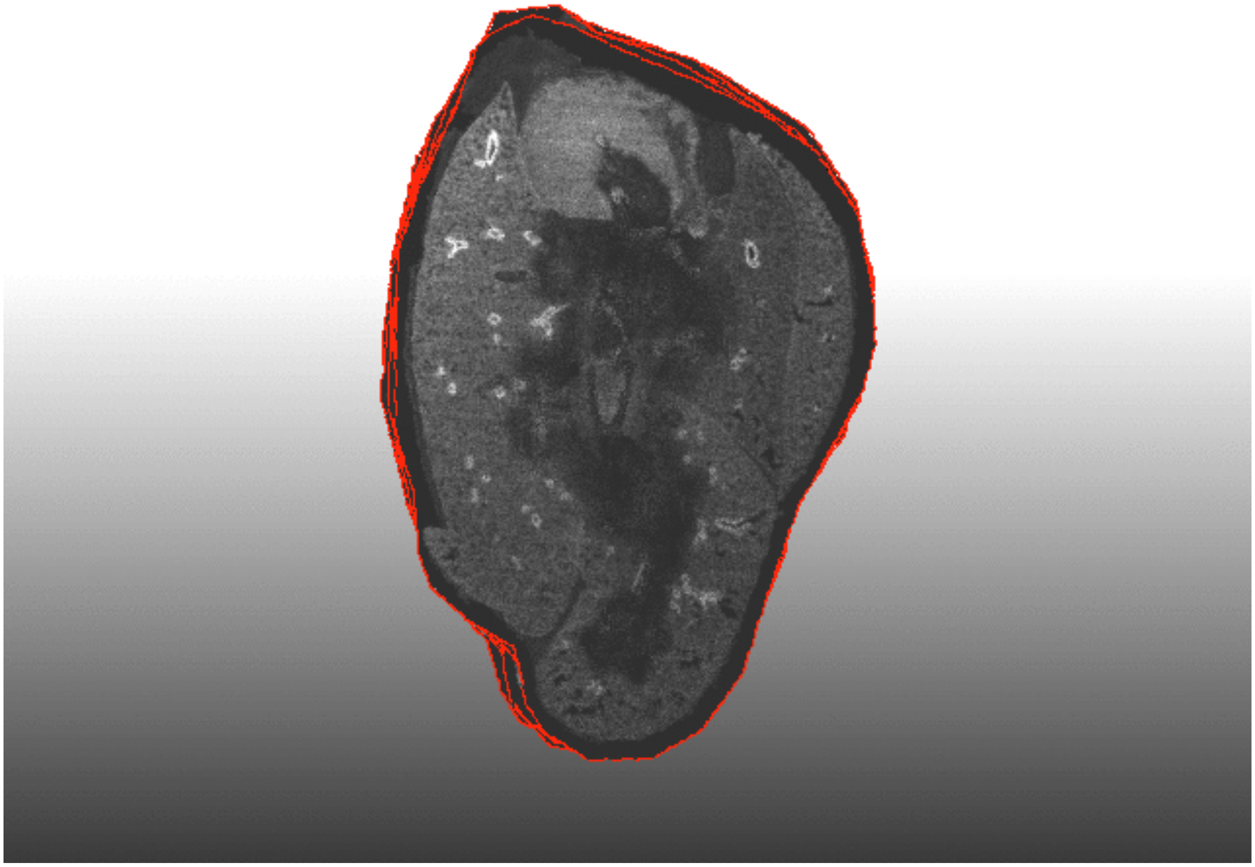

Figure S10: 3D video rendering of *m/z* 885.5 from 24 consecutive lung sections. *Z*-stack reconstruction of 24, 13 µm sections cut from the center mass of the gelatin inflated mouse lung tissue. MALDI-TOF, 50 µm spatial resolution, normalized to TIC, 360° rotation with both horizontal and vertical toggling, 30 seconds. Scale, relative intensity, and section stills are given in **Fig. S9**, spectral reference and mass channel given in **Fig. S11**.

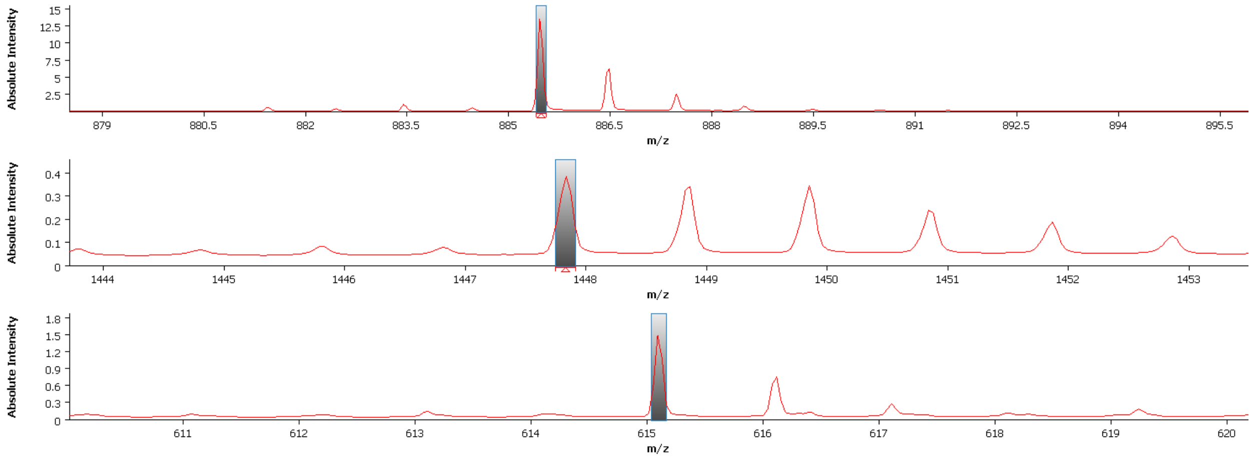

Figure S11: Mass channel windowing reference. Average spectrum (normalized, TIC) for ion channel selection: (top) *m/z* 885.5 ± 75 mDa (**Figs. 3, S9, S10**), (middle) *m/z* 1447.8 ± 80 mDa (**Figs. 3, S8**), (bottom) *m/z* 615.1 ± 65 mDa (**Figs. 3, S7**).

Table S1: MS/MS fragmentation supporting molecular assignments.

Precursor *m/z*	Assigned sum-composition species (ppm error)	Supporting MS/MS fragments[1]	Assigned primary molecular lipid species[2]
913.58049	[PI(40:4-H]$^-$ (-0.7 ppm)	629.3 [-FA 18:0(+HO)], 599.3 [-FA 22:4(-H)], 581.3 [-FA 22:4(+HO)], 419.3 [FA 18:0(+C3H5O5P)], 331.3 [FA 22:4(+O)], 283.2 [FA 18:0(+O)]	[PI(18:0_22:4)-H]$^-$
885.54886	[PI(38:4)-H]$^-$ (-1.1 ppm)	619.3 [-FA 18:0(-H)], 601.3 [-FA 18:0(+HO)], 599.3 [-FA 20:4(-H)], 581.3 [-FA 20:4(+HO)], 439.2 [FA 20:4(+C3H5O5P], 437.3 [FA 18:0(+C3H7O6P], 419.3 [FA 18:0(+C3H5O5P)], 303.2 [FA 20:4(+O)], 283.2 [FA 18:0(+O)], 259.2 [FA 20:4(-CO)]	[PI(18:0_ 20:4)-H]$^-$
883.53334	[PI(38:5)-H]$^-$ (-1.0 ppm)	601.3 [-FA 18:1(+HO)], 597.3 [-FA 20:4(-H)], 579.3 [-FA 20:4(+HO)],	[PI(18:1_20:4)-H]$^-$
881.51767	[PI(38:6)-H]$^-$ (-1.0 ppm)	625.2 [-FA 16:0(+HO)], 571.2 [-FA 22:6(-H)], 553.3 [-FA 22:6(+HO)], 463.2 [FA 22:6(+C3H5O5P)], 391.2 [FA 16:0(+C3H5O5P], 327.2 [FA 22:6(+O)], 283.3 [FA 22:6(-CO)], 255.1 [FA 16:0(+O)]	[PI(16:0_22:6)-H]$^-$
863.56505	[PI(36:1)-H]$^-$ (-0.5 ppm)	599.3 [-FA 18:1(-H)], 581.3 [-FA 18:0(-H)], 579.3 [-FA 18:0(+HO)], 419.2 [FA 18:0(+C3H5O5P)], 283.2 [FA 18:0(+O)], 281.1 [FA 18:1(+O)]	[PI(18:0_18:1)-H]$^-$
857.51770	[PI(36:4)-H]$^-$ (-1.0 ppm)	601.3 [-FA 16:0(-H)], 571.3 [-FA 20:4(-H)], 553.3 [-FA 20:4(+HO)], 439.2 [FA 20:4(+C3H5O5P)], 409.2 [FA 16:0(+C3H7O6P)], 391.2 [FA 16:0(+C3H7O5P)], 303.2 [FA 20:4(+O)], 255.2 (FA 16:0 (+O)], 241.0 [PI(241)], 417.2 [FA 18:1(+C3H5O5P)], 303.2 [FA 20:4(+O)], 281.2 [FA 18:1(+O)]	[PI(16:0_20:4)-H]$^-$
838.55959	[PS(40:4)-H] (-0.9 ppm)	751.5 [-PS(87)], 467.2 [-FA 18:0(+HO) -PS(87)], 437.3 [-FA 22:4(-H) -PS(87)], 419.3 [-FA 22:4(+HO) -PS(87)], 331.2 [FA 22:4(-CO)], 283.2 [FA 18:0(+O)]	[PS(18:0_22:4)-H]$^-$
810.52813	[PS(38:4)-H]$^-$ (-1.1 ppm)	723.5 [-PS(87], 457.2 [-FA 18:0(-H) -PS(87)], 439.2 [-FA 18:0(+HO) -PS(87)], 437.3 [-FA 20:4(-H) -PS(87)], 419.3 [-FA 20:4(+HO) -PS(87)], 303.2 [FA 20:4(+O)], 283.2 [FA 18:0(+O)]	[PS(18:0_20:4)-H]$^-$
797.53349	[PG(38:4)-H]$^-$ (0.4 ppm)	419.3 [-FA 20:4(+HO) -PG(74)], 303.2 [FA 20:4(+O)], 283.2 [FA 18:0(+O)]	[PG(18:0_20:4)-H]$^-$
795.51769	[PG(38:5)-H]$^-$ (-0.6 ppm)	483.2 [-FA 22:5(-H)/-FA 16:0(-H) -PG(74)], 465.2 [-FA 22:5(+HO)/-FA 16:0(+HO) -PG(74)], 329.2 [FA 22:5(+O)], 285.3 [FA 22:5(-CO)], 255.2 [FA 16:0(+O)]	[PG(16:0_22:5)-H]$^-$
793.50180	[PG(38:6)-H]$^-$ (-0.9 ppm)	555.3 [-FA 16:0(-H)], 537.3 [-FA 16:0(+HO)], 483.3 [-FA 22:6(-H)], 465.2 [-FA 22:6(+HO)], 391.2 [-FA 22:6(+HO) -PG(74)], 327.2 [FA 22:6(+O)], 283.2 [FA 22:6(-CO)], 255.2 [FA 16:0(+O)]	[PG(16:0_22:6)-H]$^-$
788.54397	[PS(36:1)-H]$^-$ (-0.9 ppm)	701.5 [-PS(87)], 437.3 [-FA 18:1(-H) -PS(87)], 435.2 [-FA 18:0(-H) -PS(87)], 419.3 [-FA 18:1(+HO) -PS(87], 417.2 [-FA 18:0(+HO) -PS(87)], 283.2 [FA 18:0(+O)], 281.2 [FA 18:1(+O)]	[PS(18:0_18:1)-H]$^-$
769.50187	[PG(36:4)-H]$^-$ (-0.8 ppm)	513.3 [-FA 16:0(+HO)], 483.3 [-FA 20:4(-H)], 465.3 [-FA 20:4(+HO)], 391.2 [-FA 20:4(+HO) -PG(74)], 303.2 [FA 20:4(+O)], 259.3 [FA 20:4(-CO)], 255.2 [FA 16:0(+O)]	[PG(16:0_20:4)-H]$^-$
766.53858	[PE(38:4)-H]$^-$ (-0.8 ppm)	480.3 [-FA 20:4(-H)], 462.3 [-FA 20:4(+HO)], 303.2 [FA 20:4(+O)]. 283.2 [FA 18:0(+O)], 259.3 [FA 20:4(-CO)]	[PE(18:0_20:4)-H]$^-$
764.52324	[PE(38:5)-H]$^-$ (-0.4 ppm)	478.3 [-FA 18:1(+HO)], 303.2 [FA 20:4(+O)], 281.2 [FA 18:1(+O)]	[PE(18:1_20:4)-H]$^-$
762.50744	[PE(38:6)-H]$^-$ (-0.6 ppm)	452.3 [-FA 22:6(-H)], 327.3 [FA 22:6(+O)], 283.3 [FA 22:6(-CO)], 255.2 [FA 16:0(+O)]	[PE(16:0_22:6)-H]$^-$
745.50204	PG(34:2)-H]$^-$ (-0.9 ppm)	507.3 [-FA 16:0(-H)], 489.3 [-FA 16:0(+HO)], 483.3 [-FA 18:2(-H], 465.3 [-FA 18:2(+HO)], 415.2 [-FA 16:0(+HO) -PG(74)], 391.2 [-FA 18:2(+HO) -PG(74)], 279.2 [FA 18:2(+O)], 255.2 [FA 16:0(+O)]	[PG(16:0_18:2)-H]$^-$
738.50753	[PE(36:4)-H]$^-$ (-0.5 ppm)	452.3 [-FA 20:4(-H)], 303.2 [FA 20:4(+O)], 259.2 [FA 20:4(-CO)], 255.2 [FA 16:0(+O)]	[PE(16:0_20:4)-H]$^-$

[1] Fragment identities were obtained by comparing MS/MS spectra with the list of expected peaks obtained from the online ALEX[123] lipid calculator (http://alex123.info/ALEX123/MS.php). Only fragment ions with an intensity greater than 0.05 were assigned. Fragment nomenclature is based on that proposed in *Pauling, J. K., Hermansson, M., Hartler, J., Christiansen, K., Gallego, S. F., Peng, B., Ahrends, R., Ejsing, C. S., Proposal for a Common Nomenclature for Fragment Ions in Mass Spectra of Lipids. PLOS ONE 2017, 12, e0188394.* [5]

[2] Due to the precursor ion isolation width of ±0.5 Da it is likely isobaric and isomeric precursor ions are also co-isolated and fragmented. Based on the MS/MS spectra and the assigned sum-composition formula from the high resolution MS[1]Orbitrap data we have identified the most abundant isomer for the given sum-composition species.

MATERIALS & METHODS FOR SUPPLEMENTAL EXPERIMENTS

Ion Suppression Screening

All images were captured on a Bruker solariX XR MALDI-FTICR in positive ion mode. A serial dilution of a quantitative standard lipid PC 17:0/14:1 (#LM1004, Avanti Polar Lipids, Alabaster, Alabama) was made from 1.4 nM to 1.4 µM in a solution of 1:2:0.8 parts (v:v:v) chloroform:methanol:water (CMW). Serial dilutions were spotted in 1 µL volumes on plain ITO glass, a 13 µm section of 2% gelatin, a 13 µm section of air inflated lung, and a 13 µm section of gelatin inflated lung prepared according to this method followed by NRM (10mg/mL in 1:2:0.8 CMW) application using a SunCollect matrix sprayer with the following settings: x=0.5 mm, y=2 mm, z=30.6 mm, z offset 28.0 mm, x and y speeds = medium (1), 12 total layers at speeds 1=8 µL/min, 2=10 µL/min, 3=15 µL/min, and 4+=20 µL/min. Spots on tissue were limited to the distal areas of the lung. Spot profiling of the standard was performed on a 7 T solariX XR MALDI-FTICR in positive ion mode calibrated to < 3 ppm. Spectra were collected from five different positions within the spot area, 250 shots per position at 2000 Hz with laser focus set to minimum. Spectra were converted from .baf files to .mzXML files using CompassXport (Bruker Daltonics) and imported into mMASS for analysis. A minimum detection threshold was set at 300,000 arbitrary intensity and spectra with no detectable feature for the standard were reported at 50% of threshold for graphical purposes. Average intensity and standard deviation were calculated and reported for each condition.

Image Figure Preparation Note

All MSI images were exported from flexImaging or SCiLS with color scales optimized for digital display. Figures were prepared for printed display by adjusting the brightness setting

uniformly across entire data panels (including color scales) to best represent the digital image in printed form. Native image files will be made available upon reasonable request.

SUPPLEMENTAL REFERENCES

1. Kaletaş, B. K. *et al.* Sample preparation issues for tissue imaging by imaging MS. *Proteomics* **9,** 2622–2633 (2009).
2. Chaurand, P. *et al.* Imaging Mass Spectrometry of Intact Proteins from Alcohol-Preserved Tissue Specimens: Bypassing Formalin Fixation. *ACS Publications* **7,** 3543–3555 (2008).
3. Jones, E. E., Quiason, C., Dale, S. & Shahidi-Latham, S. K. Feasibility Assessment of a MALDI FTICR Imaging Approach for the 3D Reconstruction of a Mouse Lung. *J. Am. Soc. Mass Spectrom.* **28,** 1709–1715
4. Karnati, S. *et al.* Quantitative lipidomic analysis of mouse lung during postnatal development by electrospray ionization tandem mass spectrometry. *PLoS ONE* **13,** e0203464 (2018).
5. Pauling, J. K. *et al.* Proposal for a common nomenclature for fragment ions in mass spectra of lipids. *PLoS ONE* **12,** e0188394 (2017).
